# Study of Correlation between Intestinal Microbiota and Traditional Chinese Medicine Syndrome of Patients with Colon Cancer

**DOI:** 10.1155/2022/2989456

**Published:** 2022-07-11

**Authors:** Lu-Yao Tuo, Zhi-Rui Zhang, Hong Xin, Xu Chu, Wei Xu

**Affiliations:** Department of Integrated Traditional Chinese and Western Medicine, First Affiliated Hospital of Harbin Medical University, Harbin 150001, China

## Abstract

**Objective:**

This research aims to study the material basis of the formation and specific bacteria of traditional Chinese medicine (TCM) syndrome from the characteristics of the intestinal microbiota of patients with colon cancer (CC) before and after the operation.

**Methods:**

A cross-sectional study was conducted on 84 patients with CC and 24 healthy controls. A total of 168 and 24 stool samples were collected from CC patients before and after the operation and healthy controls. DNA was extracted from 192 stool samples and then amplified using PCR. The V3-V4 high variable areas were analyzed by 16s rDNA sequencing.

**Results:**

The community diversity, in descending order, was the healthy control group and postoperative and preoperative groups of CC patients. The abundance of beneficial bacteria was postoperative group of CC patients > healthy control group > preoperative group of CC patients. Among the comparisons of the intestinal microbiota of preoperative groups of CC patients with different TCM syndromes, the community diversity in descending order was damp heat accumulation (DHA), spleen deficiency and dampness (SDD), spleen and kidney yang deficiency (SKYD), liver and kidney yin deficiency (LKYD), and deficiency of qi and blood (QBD), respectively. Specific microbiome analysis showed that the differences in the abundance of 42 taxons were statistically significant among the preoperative groups of CC patients with the five TCM syndromes and the healthy control group. While comparing the intestinal microbiota of postoperative groups with the five TCM syndromes, the community diversity in descending order is DHA, SDD, LKYD, SKYD, and QBD. Specific microbiome analysis showed that the differences in the abundance of 46 taxons were statistically significant among the postoperative groups of CC patients with the five TCM syndromes and the healthy control group. *Streptococcus* and *Streptococcus mutans* showed no statistical significance between the preoperative group and postoperative groups of CC with DHA syndrome (*P* > 0.05). Bacteroides at phylum and genus levels showed that there was no statistical significance between the preoperative group and the postoperative group of CC with SKYD syndrome (*P* > 0.05).

**Conclusions:**

Before and after surgery, with the deterioration of TCM syndrome: DHA ⟶ SDD ⟶ SKYD ⟶ LKYD ⟶ QBD, the number of beneficial bacteria in CC patients' intestines decreased while the number of pathogenic bacteria increased, and the community structure of intestinal microbiota tends to be unitized, indicating a serious intestinal microbiological disorder. After radical surgery and perioperative intervention, the intestinal microbiota diversity and community structure of postoperative CC patients were closer to those of healthy people than preoperative. However, they were still imbalanced. The intestinal microbiota of CC patients with different TCM syndromes differs significantly, which is important for understanding the pathogenesis of CC in TCM. The DHA and SKYD syndromes in CC patients before and after surgery showed significant differences in the microbial structure. *Streptococcus* and *Streptococcus mutans* were the specific species with a significant difference in CC patients with DHA syndrome, while bacteroides were the specific species in CC patients with SKYD syndrome.

## 1. Introduction

The incidence of colon cancer (CC) ranks third among malignant tumors, with an annual increase of 4.2%. Mortality and morbidity have gradually risen to fourth and fifth in the past 20 years [1–3]. The microbiota in the intestinal lumen is a highly complex microecosystem in which bacterial components account for the vast majority. Because the intestinal microbiota plays an irreplaceable role in protecting the intestinal mucosal barrier, maintaining the homeostasis of the intestinal microenvironment, and regulating immunity, it is referred to as the second human genome [4–6]. A consensus has been reached on the argument that intestinal microbiological disturbance is a major factor in CC occurrence and development [7–9].

Although surgical resection is the primary treatment for CC, several toxic side effects caused by the tumor and related treatments continue to negatively impact patients' prognosis and quality of life. Preoperative bowel preparation, anesthesia, radical surgical resection, perioperative interventions, antibiotic administration, and traumatic stress can further aggravate the microbiota imbalance, thereby affecting patients' postoperative recovery and subsequent care [10–15]. TCM (traditional Chinese medicine) dialectical treatment based on TCM syndromes is an important part of the comprehensive treatment of CC, while the clinical significance of TCM syndrome types has always been a research hotspot. Research on its material basis is the key to improving the comprehensive efficacy of TCM. However, it is also a difficult point in the research on the combination of disease and syndrome [16–20].

High-throughput 16S rDNA sequencing technology can examine the overall changes in intestinal microbiology under various pathological conditions. In this study, this technology was applied to study the changes in the species composition and community structure diversity of the intestinal microbiota in CC patients with different TCM syndromes before and after surgery. Exploring the clinical significance of CC TCM syndrome classification and the material basis of pathogenesis changes will provide an experimental basis for the rational intervention of TCM dialectical treatment of CC based on TCM syndrome types, especially after surgery.

## 2. Methods

### 2.1. Inclusion and Exclusion Criteria

#### 2.1.1. Inclusion Criteria of CC Patients

(1) Pathological biopsy was confirmed as colon adenocarcinoma; (2) aged between 40 and 80 years old; (3) live in northeast China; (4) underwent laparoscopic radical resection of CC; (5) did not receive antibiotics or microbiological agents one month before surgery; (6) did not receive neoadjuvant therapy before surgery; (7) all patients were given the same medication regimen after surgery, fasting for three days, and complete parenteral nutrition support; and (8) meet the criteria of TCM clinical diagnosis classification.

Patients meeting one or more of the following criteria were excluded: (1) history of other cancers or inflammatory bowel; (2) received invasive treatment within the last three months; (3) eat only vegetarian food; (4) received antibiotic, microbiological agents, or targeted therapy one month before admission; (5) received neoadjuvant therapy before surgery; (6) have a mental illness or unable to participate in and complete clinical studies in a standardized manner; (7) pregnant or lactating women; (8) incomplete clinical data; and (9) others.

#### 2.1.2. Inclusion Criteria of the Healthy Control Group

(1) no personal or family history; (2) all first-degree relatives were negative for CC; (3) fiber colonoscopy result is negative; and (4) did not receive antibiotics or microbiological agents one month before colonoscopy.

### 2.2. Participants and Study Design

From March 2019 to March 2020, 84 CC patients were recruited in the Department of Colorectal Surgery at the First Affiliated Hospital of Harbin Medical University, Heilongjiang Province. All patients signed informed consent. This study was approved by the Ethics Committee of the First Affiliated Hospital of Harbin Medical University (No. 201931) and conducted following the principles stipulated in the Helsinki Declaration. This study has been registered in the North American Clinical Trial Registry (NCT03892252).

According to the National Comprehensive Cancer Network (NCCN) clinical guidelines (2021, version 1), researchers must fill in all eligible patients' baseline data, including demographic data, tumor location, pathology, genotyping, and tumor markers. Clinical data are retained in a questionnaire format to verify the original data. Before and after the operation, two senior TCM physicians conducted an assessment and grouping of TCM syndromes according to the “Standards of the Chinese Society of Chinese Medicine: Guidelines for TCM Diagnosis and Treatment of Tumors” (2009, version 1), including five TCM syndromes, namely, damp heat accumulation (DHA), spleen deficiency and dampness (SDD), spleen and kidney yang deficiency (SKYD), liver and kidney yin deficiency (LKYD), and qi and blood deficiency (QBD). Patients during hospitalization were chosen to improve participant compliance, and standardized treatment procedures were used before and after the operation to minimize interference from other factors.

### 2.3. Queue Design

**Figure 1:**
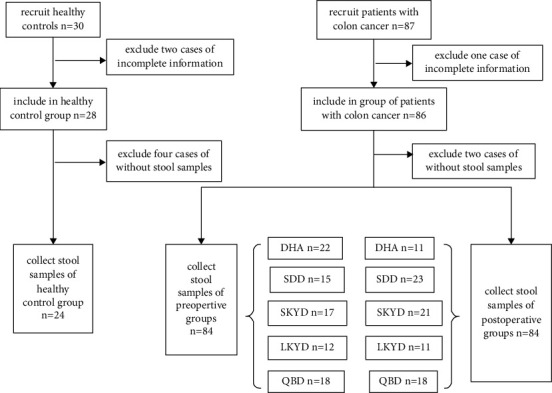
Queue design, DHA: damp heat accumulation, SDD: spleen deficiency dampness, SKYD: spleen and kidney yang deficiency, LKYD: liver and kidney yin deficiency, QBD: Qi and blood deficiency.

The queue design of this study was shown in Figure 1.

### 2.4. Sample Collection Methods

#### 2.4.1. Sample Collection

After admission before bowel preparation and the first bowel movement after the operation, 5 g stool samples were collected for CC patients. Stool samples from the healthy control group were collected in an outpatient clinic. All samples were sent to a −80°C refrigerator for storage two hours after collection.

#### 2.4.2. DNA Extraction

According to the manufacturer's instructions, DNA extraction is performed using the Fast DNA Spin Kit for Soil (MP Biotechnology, United States). Monitor DNA concentration and purity on a 1% agar gel. The Nanodrop 2000 (10x Genomics, USA) detects whether the quality of genomic DNA is qualified (concentration ≥ 20 ng/*μ*L, total ≥ 500 ng, OD260/280 = 1.8 to 2.0).

#### 2.4.3. PCR Amplification

High-fidelity PCR amplification was used to amplify the qualified sample test area, and three repeated experiments were set up with standard bacterial genome DNA Mix as a positive control. The target area is the 16S V3-V4 region. Primer F = Illumina connector sequence 1 + CCTACGNGGCWGCAGAG, Primer R = Illumina connector sequence 2 + GACTACHVGGTATTATTAATCC, Illumina connector sequence 1 + AATGATCGACCACCACCACCACCACXXXXXTCGCGATGATAAGAGACAG, Illumina connector sequence 2 + CTGCGCGATGTCTTATACATACCTCCGACCCACCACCACCACXXXXXXATCTCTCTCTCTCTG, XXXXXXXXXX is the sequence of labels that identify the sample.

#### 2.4.4. 16sr DNA Sequencing

To purify the product and obtain the original library, we add an equal volume (Beckman Coulter, USA) of nucleic acid purification magnetic beads to each sample. Based on the preliminary quantitative results of the agar gel electrophoresis, appropriately dilute the sample library concentration with the corresponding index label, using the Invitrogen Qubit 3.0 Spectrophotometer (Thermo Fisher Scientific, USA) to quantify the library and mix the samples accurately. The proportion corresponds to the sequencing flux requirements of different samples (molar ratio).

### 2.5. Bioinformatics Analysis

#### 2.5.1. Data Processing and Quality Control

The data of each sample are distinguished according to the index sequence, and the extracted data is saved in fastq format. Each sample has two files, fq1 and fq2. After base identification and error filtering, a raw read that can be used for analysis is finally obtained. TrimGalore, FLASH2 Mothur, and Research software are used to remove, splice, and optimize sequences to obtain optimized sequences with higher quality and reliability (clean read) (Supplementary Table S1 for detailed data) [21, 22].

#### 2.5.2. Rank-Abundance Curves and Rarefaction Curve

Rank-abundance curves explain the richness and uniformity of the species contained in the sample. Rarefaction curves are constructed from the number of randomly selected sequences and the number of OTUs they can represent, which can visually display the difference in species richness between samples and can also be used to evaluate whether the sequencing amount of samples is reasonable. The two diagrams were made by the R language tool.

#### 2.5.3. Analysis of Similarities

Anosim analysis is a nonparametric test method based on the permutation test and rank-sum test, which is used to test whether the difference among groups is significantly greater than that within groups to judge whether the grouping is meaningful. We use the ANOSIM function in R's vegan package for analysis, and the distance algorithm uses “bray.”

#### 2.5.4. Alpha Diversity Analysis

The species richness index (Chao1) estimates the total number of species in the sample. The Shannon index is used to reflect sample diversity. Based on the Kruskal–Wallis rank-sum test, the difference analysis of the diversity index among groups was carried out, and the *P* value <0.05 was used as the difference significance screening threshold. The Bonferroni method was used to modify the multihypothesis *P* value test (FDR). The two indexes are executed by the Mothur software.

#### 2.5.5. Differential Microbiome Analysis

ANOVA variance analysis identifies species in multiple groups with significant differences at each classification level. The default threshold is 0.05.

#### 2.5.6. Specific Microbiome Analysis

LefSe analysis is based on significant differences in species, using a paired Wilcoxon rank test for difference analysis among groups. Linear discriminant analysis (LDA) is used to evaluate the effect size of each species with significant differences. LDA score is a logarithmic transformation with log as the base 10 by default. The larger the absolute value, the easier it is to distinguish groups. |LDA| > 2 and *P* < 0.05 are used as the difference screening threshold to obtain the species most likely to explain the difference among groups.

#### 2.5.7. Functional Predictive Analysis

We compared the sequencing data with the Greengenes database with known metabolic functions, COG orthology, and KEGG pathway were obtained. Using Welch's *T*-test to compare the relative abundance of the functions (COG/KEGG) of the two groups to find out the functions with significant differences in the two groups. *P* value <0.05 was used as the threshold for the significance of the difference.

#### 2.5.8. Random Forest Analysis

We can find the key OTU to distinguish the differences between groups through random forest (RF) analysis. The random sampling method with replacement is used to select samples, and then the independent and dependent variables are randomly selected. The average impurity and Gini impurity are reduced according to the average accuracy of data splitting, and different classification trees are created [23]. The Gini index is used to calculate the impact of each variable on the heterogeneity of the observed value of each classification tree node [24]. Our study modeled and analyzed the relative abundance data at the genus level among preoperative groups of CC patients with the five TCM syndromes and the healthy control group.

### 2.6. Statistics

The statistical software (SPSS 22.0) was used to analyze the differences in microbiological data among groups. The measurement data is expressed in *x* ± *s*. A *T*-test represents the comparison between two separate samples. The differences are considered statistically significant (*P* < 0.05), significant (*P* < 0.01), and nonstatistical (*P* > 0.05).

## 3. Results

### 3.1. General Situation

The patients in this experiment lived in Heilongjiang Province, China. Their diet was characterized by high fat, sugar, and salt due to their living habits and climate. All clinical data and parameters are summarized in Tables 1 and 2.

### 3.2. Sequencing Depth

**Figure 2:**
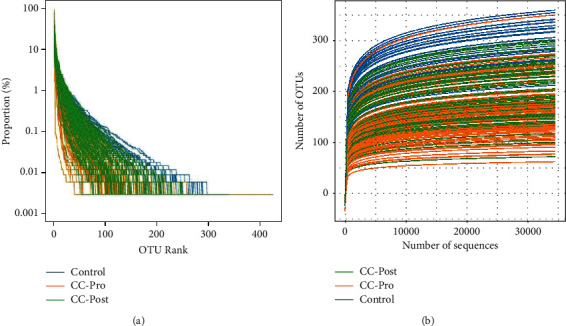
(a): Rank-abundance curve, a commonly used visual analysis method to assess species diversity. (b): rarefaction curve, through comparing the dilution curves of different samples, the difference in species richness among samples and whether the amount of sequencing of samples is reasonable can be visually displayed.

The number of OTU clusters out of 192 samples in this study was 2213. The rank abundance curve (Figure 2(a)) showed that the pattern of the postoperative group of CC patients was similar to that of healthy controls, but the latter had a longer right tail, indicating that the healthy people had a higher abundance and more flora distribution than that of the postoperative group. As the curve flattens out (Figure 2(b)), extracting more data produces only a small number of new OTUs. This indicates that the amount of sequencing data for the sample is reasonable.

### 3.3. Comparison of the Microbiome among Preoperative and Postoperative CC Patients and Healthy Control Group

**Figure 3:**
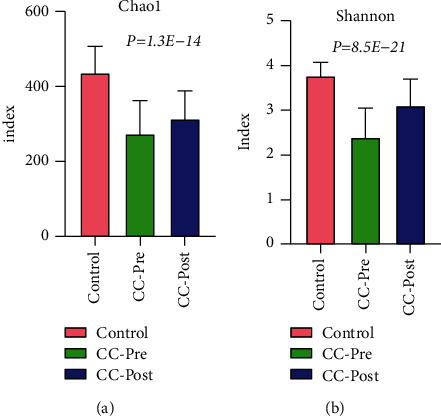
(a): The index of Chao1 was used to estimate the total number of species in the sample, the larger the value, the more species. (b): the index of Shannon was used to estimate the community diversity of the sample, the larger the Shannon value, the higher the community diversity. The abscissa represents different groups, the ordinate represents the community diversity index of the group, different colors represent the healthy control group, the CC preoperative patient group, and the CC postoperative patient group, respectively.

The community richness (Chao1) and diversity (Shannon) in descending order were the healthy control group, CC-post, and CC-pre group (Figures 3(a) and 3(b)) (*P* < 0.05). Detailed characteristics of community diversity for each sample can be found in Supplementary Table S2. ANOSIM analysis showed that the R-value and significance were 0.23 and 0.0001, respectively. The dominant and differential bacterial species at phylum, genus, and species levels of the three groups are revealed in Supplementary Table S3.

### 3.4. Analysis of the Microbiome Structure of Preoperative Groups of CC Patients with Different TCM Syndromes

**Figure 4:**
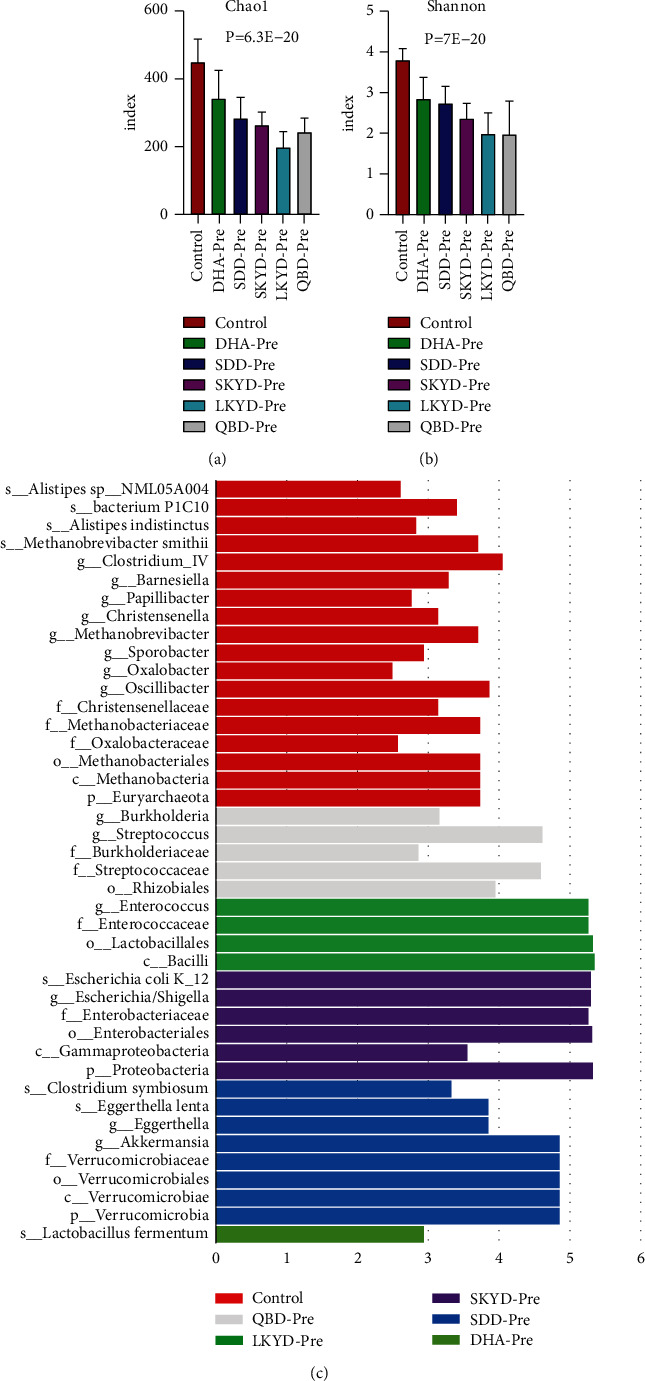
(a) and (b) represent the Chao1 index and Shannon index of the group of CC preoperative patients with the five TCM syndromes, respectively. (c) The LDA score obtained by linear regression analysis (LDA). The threshold of linear discriminant analysis score was set at 2. The larger the LDA score is, the greater the differences among six groups are. Different colors represent preoperative groups of CC patients with the five TCM syndromes and healthy controls, respectively. This figure shows not only the specific species of each group but also the species with statistically significant differences among the groups.

The indexes of Chao1 and Shannon of the healthy control group were the highest. The descending order is DHA, SDD, SKYD, LKYD, and QBD (Figures 4(a) and 4(b)) (*P* < 0.05). ANOSIM analysis showed the R-value and significance were 0.44 and 0.0001, respectively. LefSe analysis yielded 84 taxons with significant differences in relative abundance among groups. Furthermore, ANOVA analysis indicated that 18, 1, 8, 6, 4, and 5 taxons have statistically significant differences among the healthy control group and the preoperative group of DHA, SDD, SKYD, LKYD, QBD (Supplementary Table S4 and Figure 4(c)).

### 3.5. Analysis of the Microbiome Structure of Postoperative Groups of CC Patients with Different TCM Syndromes

**Figure 5:**
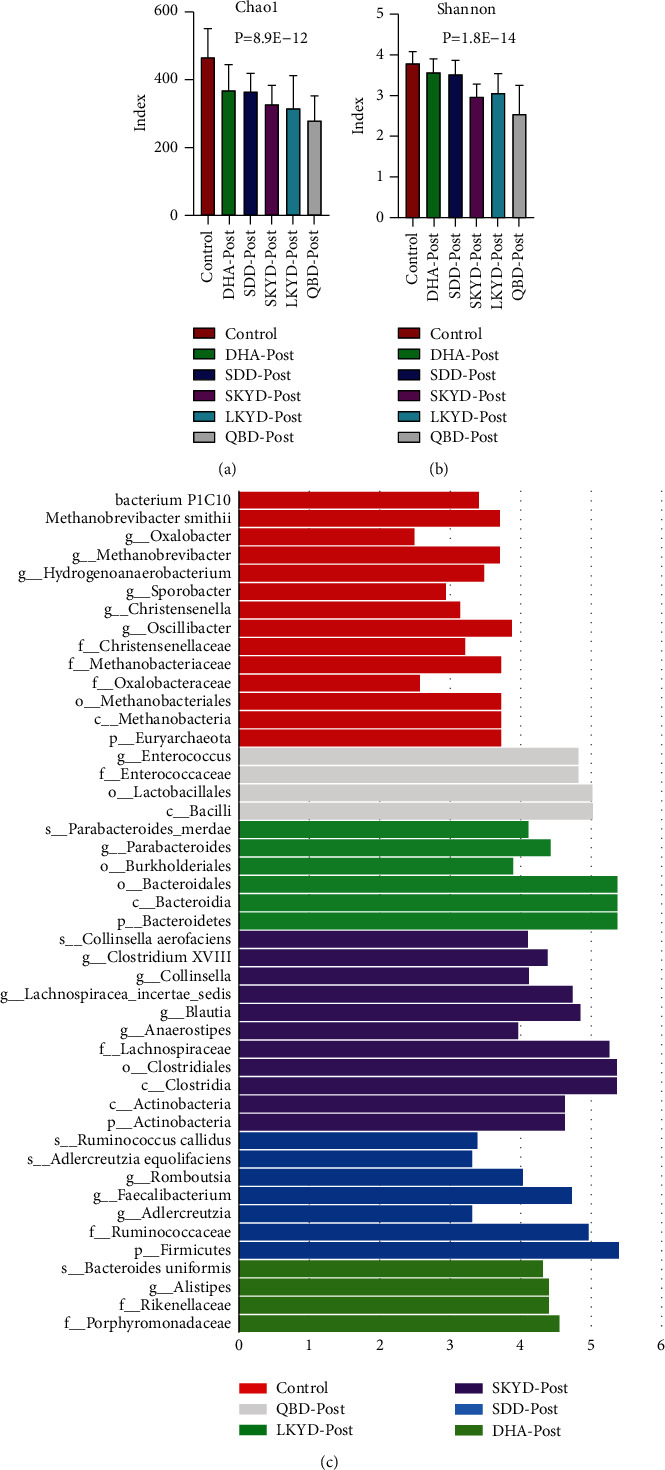
(a) and (b) represent the Chao1 index and Shannon index of the group of CC postoperative patients with the five TCM syndromes, respectively. (c) The LDA score obtained by linear regression analysis (LDA). The threshold of linear discriminant analysis score was set at 3, the larger the LDA score is, the greater the differences among six groups are. Different colors represent postoperative groups of CC patients with the five TCM syndromes and healthy controls, respectively. This figure only shows not the specific species of each group but also the species with statistically significant differences among the groups.

The index of Chao1 and Shannon of the healthy control group were the highest, descending order is DHA, SDD, LKYD, SKYD, and QBD, respectively (Figures 5(a) and 5(b)) (*P* < 0.05). ANOSIM analysis showed that the R-value and significance were 0.46 and 0.0001, respectively. According to LefSe analysis, 81 taxons with significant differences in relative abundance among groups were obtained. Furthermore, ANOVA variance analysis indicated that among 81 taxons, there are 14, 4, 7, 11, 6, and 4 taxons with statistically significant differences among the healthy control group and the postoperative group of DHA, SDD, SKYD, LKYD, QBD, and healthy control group, respectively (Supplementary Table S5 and Figure 5(c)).

### 3.6. Analysis of Bacterial Community Structure between the Preoperative and Postoperative Groups of CC Patients with DHA Syndrome

**Figure 6:**
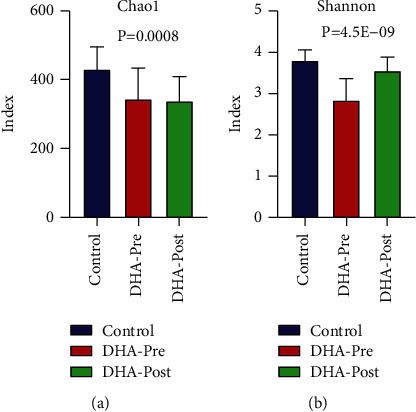
(a) and (b) represent the Chao1 index and Shannon index of the CC preoperative patient group and CC postoperative patient group with DHA syndrome, respectively.

The index of Chao1 and Shannon of DHA-pre were higher than that of DHA-post (Figures 6(a) and 6(b)) (*P* < 0.05). ANOSIM analysis showed that the R-value and significance were 0.32 and 0.0001, respectively. The dominant and statistically significant taxons are revealed in Table 3.

### 3.7. Analysis of Bacterial Community Structure between the Preoperative and Postoperative Groups of CC Patients with SKYD Syndrome

**Figure 7:**
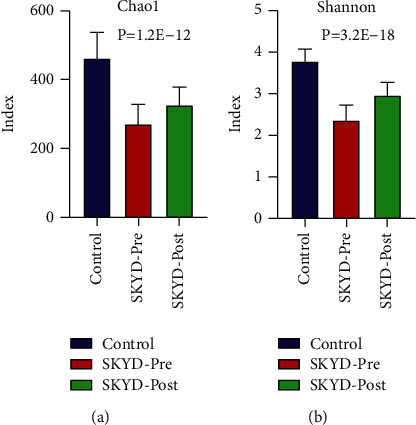
(a) and (b) represent the Chao1 index and Shannon index of the CC preoperative patient group and CC postoperative patient group with SKYD syndrome, respectively.

The index of Chao1 and Shannon of SKYD-pre was higher than that of the SKYD-post group (Figures 7(a) and 7(b)) (*P* < 0.05). ANOSIM analysis showed that the R-value and significance were 0.32 and 0.0001, respectively. The dominant and statistically significant taxons are displayed in Table 4.

### 3.8. Prediction of Intestinal Microbiota in the Preoperative Groups of CC Patients with Different CM Syndrome

**Figure 8:**
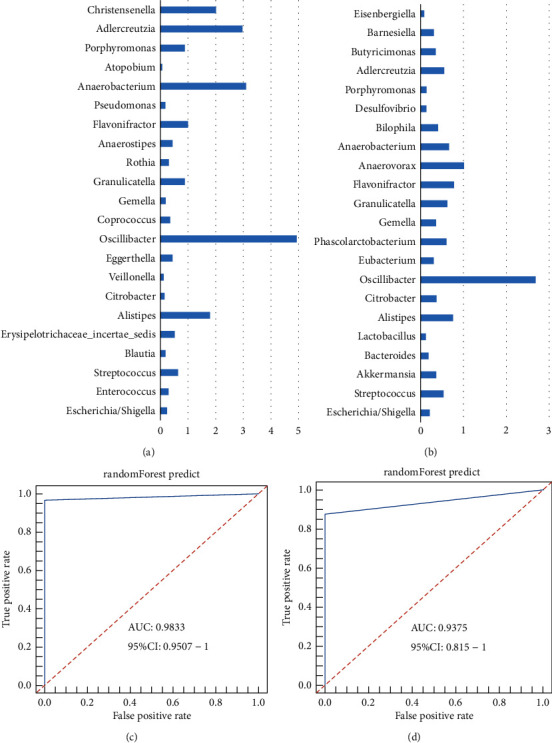
(a): the first 20 variables in the random forest model for CC preoperative patients in the validation cohort; (b): the first 20 variables in the random forest model for CC preoperative patients with DHA syndrome in the validation cohort; (c): the ROC curve of the random forest model of CC preoperative patients in the validation cohort; and (d): the ROC curve of the random forest model of CC preoperative patients with DHA syndrome in the validation cohort.

The RF model was established to predict the markers of CC patients with different CM syndromes in the experimental cohort, and then its accuracy was evaluated in the validation cohort. The ROC curve was used to quantify its diagnostic potential. Figures 8(a) and 8(b) demonstrate a bar graph of the top 20 bacterial genera with the most variables at the genus level (average decreasing Gini > 1). It was found that Alipites, Oscillibacter, Flavonifractor, Anaerobacterium, Christensenella, and Adlercreutzia can distinguish CC preoperative patients from healthy people (AUC = 0.9833, 95% CI: 0.9507-1, Figure 8(c)), and Oscillibacter, Anaerovorax, and Christensenella can distinguish CC preoperative patients with DHA syndrome from other CM syndromes (AUC = 0.9375, 95% CI: 0.815-1, Figure 8(d)). However, this cannot distinguish the other four TCM syndromes of CC preoperative patients from the healthy people (AUC = 1, 95% CI: 1-1). Thus, it has not been shown.

### 3.9. Feature Prediction

**Figure 9:**
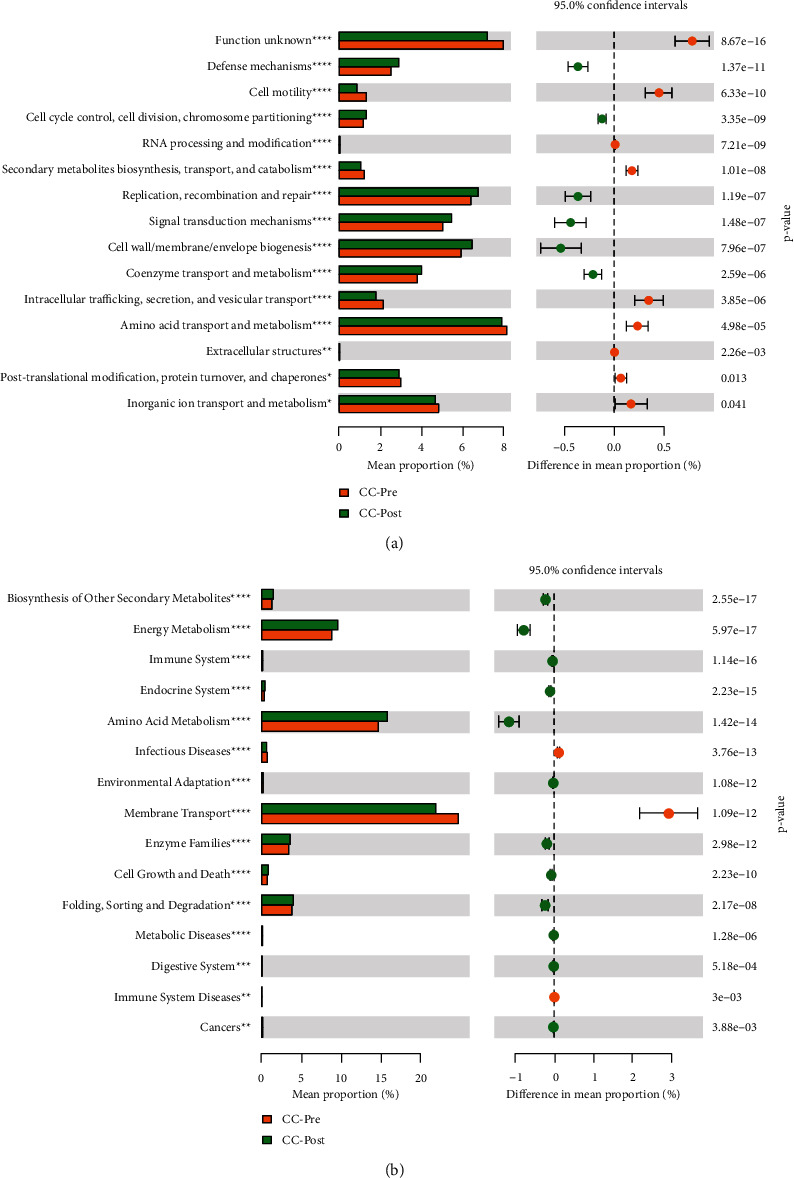
(a) The bar chart of COG function annotation using the *T*-test between preoperative and postoperative CC patient groups. (b) The bar chart of the involved enrichment metabolic pathway according to KEGG databases using the *T*-test between preoperative and postoperative CC patient groups.

**Figure 10:**
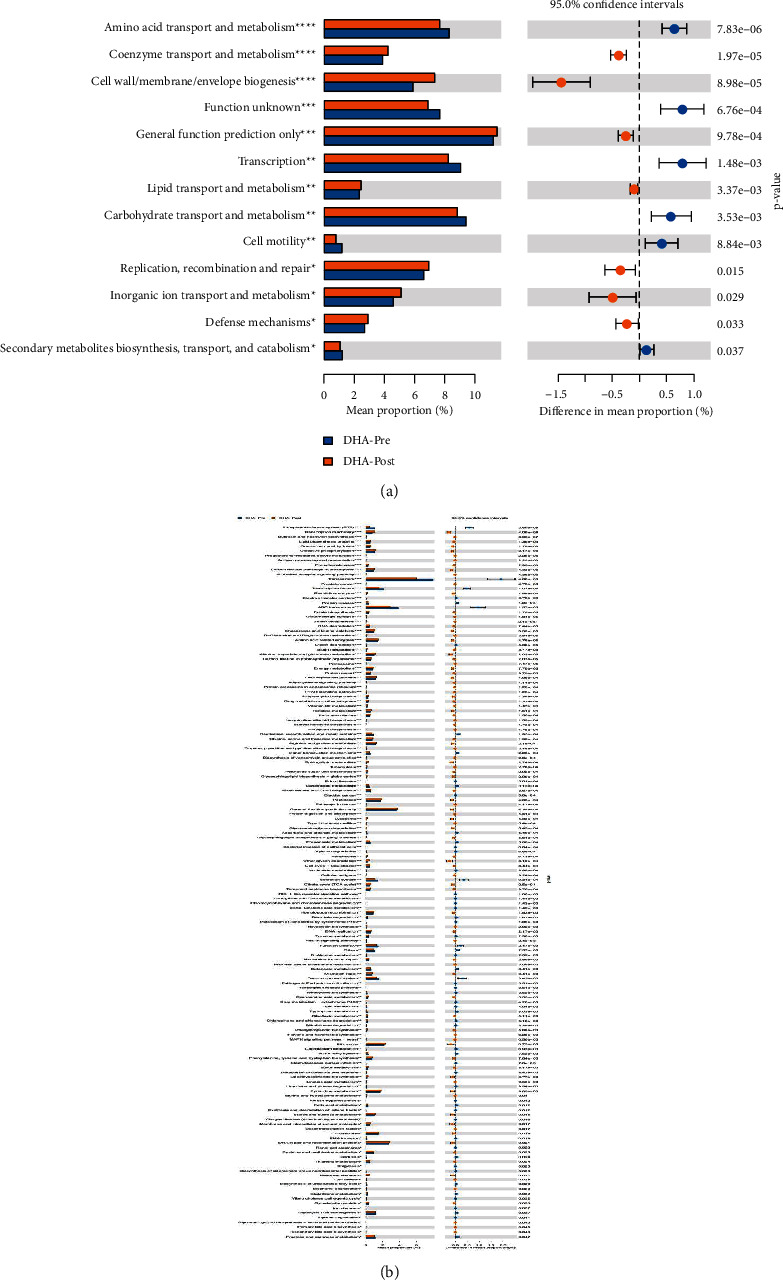
(a) The bar chart of COG function annotation using *T*-test between preoperative and postoperative CC patient groups with DHA syndrome type. (b) The bar chart of the involved enrichment metabolic pathway according to KEGG databases using the *T*-test between preoperative and postoperative CC patients Groups with DHA syndrome type.

**Figure 11:**
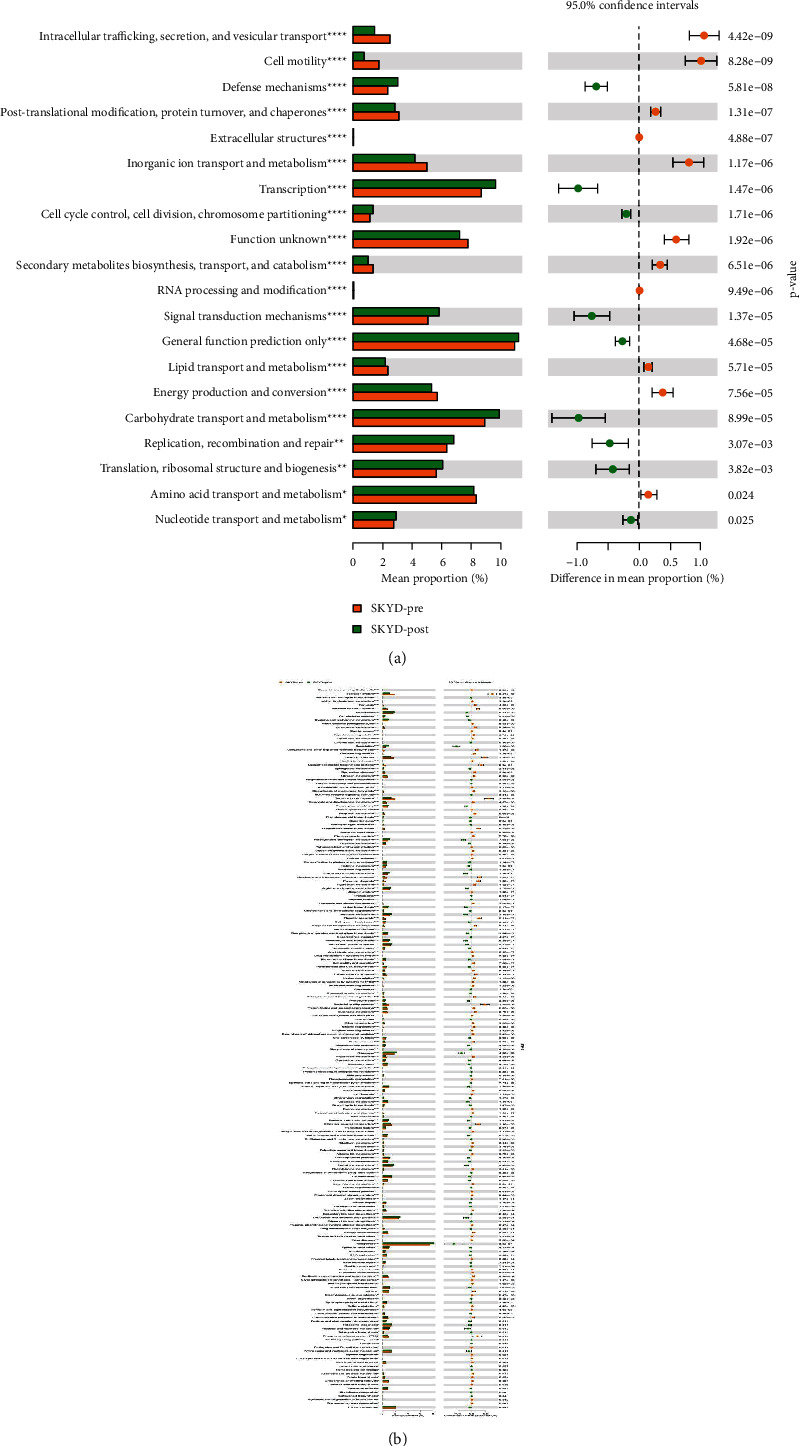
(a) The bar chart of COG function annotation using the *T* test between preoperative and postoperative CC patient groups with SKYD syndrome type. (b) The bar chart of the involved enrichment metabolic pathway according to KEGG databases using the *T* test between preoperative and postoperative CC patient groups with SKYD syndrome type.

The PICRUST software was used to predict the function of 16S rRNA sequencing data. The *T*-test was used to analyze the results of homologous clustering gene (COG) function annotation and the metabolic pathways involved in the Kyoto encyclopedia of genes and genomes (KEGG) database. The results of COG function annotation showed that there were fourteen gene functions with significant differences, such as amino acid, inorganic ion and coenzyme transport, and metabolism (Figure 9(a)). The differential metabolic pathways involved in the above functions mainly include membrane transport, biosynthesis of other secondary metabolites, amino acids, and energy metabolism between preoperative and postoperative CC patients (Figure 9(b)). According to the same method, we found twelve gene functions with significant differences in amino acid, carbohydrate, lipid, inorganic ion transport, and metabolism. Eight central enrichment metabolic pathways with a significant difference include pyrimidine, cysteine and methionine, starch and sucrose, arginine and proline metabolism, DNA repair and recombination proteins, glycolysis/gluconeogenesis, chromosome, and membrane transport in preoperative and postoperative CC patients groups with DHA syndrome type (Figures 10(a) and 10(b)). Nineteen gene functions with significant differences include nucleotide transport and metabolism, energy production, and conversion. Eight primary enrichment metabolic pathways with a significant difference include peptidases, cysteine and methionine metabolism, glyoxylate and dicarboxylate metabolism, porphyrin and chlorophyll metabolism, methane metabolism, alanine, aspartate and glutamate metabolism, pentose phosphate pathway, and purine metabolism in preoperative and postoperative CC patient groups with SKYD syndrome type (Figures 11(a) and 11(b)).

## 4. Discussion

With the transformation of the treatment model of malignant tumors, precision and individualization have become the key to improving CC diagnosis and treatment [25, 26]. Intestinal microbiota play a vital role in maintaining intestinal stability [27–29]. Changes in the composition of the host intestinal microbial community affect the barrier function of intestinal epithelial cells, host immunity, and inflammatory response, thereby affecting the occurrence and outcome of CC [30–32]. TCM treatment is crucial in the postoperative recovery of CC patients. Therefore, our team linked CC, intestinal microbiota, and TCM syndrome together, trying to analyze the material basis of TCM syndrome types in CC patients from the perspective of intestinal microbiota [33].

CC treatment can be comprehensive based on operation, preoperative bowel preparation, anesthesia, tumor resection, parenteral nutrition, and postoperative preventive antibiotics will all affect the changes in the structure and abundance of the flora [34–36]. Therefore, under the same conditions as the ERAS (enhanced recovery after surgery) model, we collected stool samples before the preoperative bowel preparation and the first bowel movement after the operation to reduce the interference of other factors on the intestinal microbiota. The study of intestinal microbiota must pay attention to the physiological differences caused by age, gender, genetics, diet, geographical location, and the competition of specific strains in different flora classifications [37–40]. The smaller the geographic area, the higher the accuracy of the random forest model, so we only selected the residents of Heilongjiang Province in China [41].

We supposed that the structure of the intestinal microbiota of CC postoperative patients would gradually become normal, and the flora changes after the operation would be more conducive to explaining the importance and necessity of tumor removal and timely and sustained intervention and treatment. To validate this viewpoint, we compared bacterial diversity and beneficial bacterial abundance among preoperative and postoperative CC patients and a healthy control group. The results in Figure 2(b) showed that the descending order of community diversity is the healthy control group > postoperative group > preoperative group, indicating that the intestinal homeostasis of CC postoperative patients was still in a state of imbalance. The descending order of the number and abundance of beneficial bacteria is CC postoperative patient group > healthy control group > CC preoperative patient group, indicating that after tumor resection, nutritional support, and the preventive use of antibiotics, the beneficial bacteria have recovered and were higher than those of the healthy control group. Thus, it also explains the necessity of tumor resection and rationalized treatment after surgery.

Dialectical treatment is the core principle in the treatment of CC in TCM. According to “Tumor TCM Diagnosis and Treatment Guidelines,” we divide CC patients into five groups: DHA, SKYD, LKYD, SDD, and QBD. We analyzed the material basis of TCM syndrome classification from the perspective of intestinal microbiota and changes in the intestinal microbiota of five TCM syndrome types of CC patients before and after surgery through 16S rDNA sequencing.

We assumed that the preoperative and postoperative CC patients with the five TCM syndromes all have their own specific flora different from other TCM syndromes and the relatively stable flora structure of the syndrome itself. We performed the comparison to validate our point of view, as displayed in Supplementary Table S4. The results showed that the community structure and specific and differential flora of CC preoperative patients with TCM syndrome were significantly different and statistically significant at different levels of bacterial classification. Despite the intervention of short-term factors such as tumor resection and complete parenteral nutrition, the community structure and specific flora of CC postoperative patients with TCM syndrome still had significant differences at the level of phylum, genus, and species, which in turn proved the rationality of interpreting dialectical parting from the perspective of intestinal microbiota. This indicates a correlation between TCM syndrome and the intestinal microbiota.

According to the horizontal comparison of the five TCM syndromes of CC patients before and after the operation, we further wanted to clarify the biomarkers of a specific TCM syndrome. We assumed that the existence of a specific flora structure could serve as the material basis for TCM syndrome. Thus, we focused on the differences in flora before and after the operations of CC patients with DHA and SKYD syndrome, which had the most significant number of cases and at different stages of TCM pathogenesis, and made a vertical comparison to analyze the specific and differential species.

According to the LDA method, we used |LDA| > 2 and *P* < 0.05 as screening the threshold to obtain the dominant species of each group, and then the nonparametric Kruskal–Wallis rank-sum test was used to screen the species with significant differences in relative abundance among groups. According to Table S4, the dominant species with significant differences in CC preoperative patients with DHA syndrome was Lactobacillus ferment (0.017%), while those of CC postoperative patients were Porphyromonadaceae, Rikenellaceae, Alitipes, and Bacteroides uniformis (*P* < 0.05).

In the comparative analysis of specific species of CC patients with DHA syndrome before and after surgery, as listed in Table 3, *Streptococcus* (8.97% Vs. 3.98%) and *Streptococcus* mutans (3.42% Vs. 1.48%) have no significant difference between the preoperative and postoperative groups (*P* > 0.05). However, there is a significant change compared with their abundance in the healthy control group (FDR < 0.05), so it is necessary to conduct further research on whether it is indeed a specific species of DHA syndrome. According to research, *Streptococcus* in CC tumor tissues has been linked to an inflammatory response that alters the intestinal microenvironment to promote progression [42–44]. In addition to the study of species correlation, we also performed functional predictions on specific flora and found that they are closely related to pyruvate metabolism, purine metabolism, and aminoacyl-tRNA biosynthesis.

To verify the dominant species with significant differences and specific microbiome in CC patients with SKYD syndrome before and after surgery, we performed the vertical analysis as revealed in Table 4. The dominant species with significant differences in preoperative patients with SKYD syndrome were Proteobacteria, Gammaproteobacteria, Enterobacteriales, Enterobacteriaceae, *Escherichia*/*Shigella*, and *Escherichia coli* K-12. Concurrently, Actinobacteria, Actinobacteria, Blautia, Clostridia, Clostridiales, Lachnospiraceae, Anaerostipes, Collinsella, *Clostridium* XVIII, Lachnospiracea incertae sedis, and Collinsella aerofaciens were the dominant species with significant differences in postoperative patients. (FDR < 0.05) The abundance of Proteobacteria in patients with SKYD syndrome after surgery decreased significantly. Many studies have revealed that Proteobacteria can be used as a microbial marker for intestinal microbiome disorder, which can also reflect the degree of microbiological dysbiosis or unstable community structure [32, 45]. Microbiological imbalance during metabolic disorder is often accompanied by an increase in Proteobacteria [46].

While there was no significant difference in the relative abundance of Bacteroides at the phylum and genus levels between preoperative and postoperative (*P* > 0.05, Table 4), this can be investigated further as a microbiota marker of CC patients with SKYD syndrome. Studies have shown that Bacteroides can metabolize the production of short-chain fatty acids, which play an important role in suppressing inflammation and cell carcinoma [47, 48]. The enterotoxin-producing Bacteroides fragilis secretes toxic products that can promote the decomposition of E-cadherin and induce Th17/IL-17 inflammation, while the enterotoxin-producing Bacteroides fragilis has no such effect [49–51]. Therefore, Bacteroides and their metabolites have a dual role. We performed functional predictions and found that most COG functions and KEGG pathways in CC patients with SKYD syndrome were manifested in coenzyme transport and metabolism and cell wall/membrane/envelope biogenesis.

Although this study identified specific biomarkers for CC patients with the DHA and SKYD syndromes, larger samples, multicenter designs, animal experiments, and novel research techniques are still required to investigate the potential causal mechanisms among different TCM syndromes of CC patients. Based on data support from clinical trials for Bacteroides and *Streptococcus*, we will investigate the mechanism of single bacteria to improve the correlation between TCM syndromes, intestinal microbiota, and CC.

## Figures and Tables

**Table 1:** Baseline characteristics of preoperative CC patients with different TCM syndromes.

Groups	Exploration cohort *n* = 68						Validation cohort *n* = 40					
	DHA	SDD	SKYD	LKYD	QBD	control	DHA	SDD	SKYD	LKYD	QBD	control
	*n* = 14	*n* = 9	*n* = 11	*n* = 8	*n* = 12	*n* = 14	*n* = 8	*n* = 6	*n* = 6	*n* = 4	*n* = 6	*n* = 10
M/F	10/4	5/4	9/2	2/6	5/7	7/7	4/4	4/2	5/1	1/3	3/3	5/5
Age (mean ± SD)	62.07 ± 8.21	60.78 ± 6.63	64.55 ± 8.57	64.63 ± 3.81	68.75 ± 3.91	65.5 ± 4.42	66.5 ± 7.09	63.02 ± 7.14	64.52 ± 7.01	62.82 ± 6.91	67.28 ± 7.15	66.02 ± 4.61
U/R	7/7	4/5	5/6	4/4	7/5	7/7	5/3	3/3	3/3	2/2	3/3	4/6
L/R	8/6	5/4	6/5	5/3	6/6	—	4/4	3/3	3/3	2/2	3/3	—
TNM												
I	0	0	0	0	0		0	0	0	0	0	
II	10	5	2	1	3	—	6	2	1	1	1	—
III	2	2	7	5	3		1	1	4	2	1	
IV	2	2	2	2	6		1	3	1	1	4	

**Table 2:** Summary of clinical data of post operative CC patients with different TCM syndrome types.

Groups	Control	DHA	SDD	SKYD	LKYD	QBD
	*n* = 24	*n* = 11	*n* = 23	*n* = 21	*n* = 11	*n* = 18
M/F	12/12	6/5	15/8	12/9	10/1	9/9
Year	64.75 ± 6.946	64.818 ± 6.676	64.783 ± 4.542	65.857 ± 6.981	65.363 ± 6.185	66.611 ± 3.760
U/R	12/12	5/6	9/14	11/10	6/5	11/7
L/R	—	7/4	12/11	7/14	8/3	13/5
TNM						
I		0	0	0	0	0
II	—	7	12	3	2	4
III		3	7	8	3	6
IV		1	4	10	6	8

M/F, male/female; U/R, urban/rural; L/R, tumor location of left/right colon.

**Table 3:** The dominant and statistically significant taxons at phylum, genus, and species levels among preoperative and postoperative group of CC patients with DHA syndrome and healthy control group.

Taxon	Mean in control	Mean in DHA-pre	Mean in DHA-post	FDR	P.adj
Phylum					
Bacteroidetes	0.224690292	0.033744079	0.488658782	9.1646E-09	1.80402E-10
Proteobacteria	0.071796062	0.222447435	0.056386952	0.001419252	0.001615255
Firmicutes	0.598046255	0.646722388	0.399585386	0.014170736	0.001648537
Genus					
*Bacteroides*	0.125269574	0.014130366	0.29043276	1.30153E-05	2.37624E-08
*Alistipes*	0.02965069	0.002744311	0.063196957	0.028741953	0.000184157
*Streptococcus*	0.010998124	0.089651007	0.033815291	0.028741953	0.061187423
Specics					
*Bacteroides caccae*	0.00785753	0.000279574	0.013796723	0.058326175	0.000515622
*Parabacteroides merdae*	0.006635382	0.001684039	0.015730005	0.058326175	0.00046149
*Bacteroides uniformis*	0.022079658	0.002706067	0.041260932	0.058326175	0.00097691
*Bacteroides stercoris*	0.010137422	0.0022722	0.038085391	0.088483618	0.002386112
*Bacteroides plebeius*	0.001231819	0.000486617	0.026396025	0.127533981	0.007435125
*Escherichia coli* K-12	0.045219479	0.145393092	0.024468018	0.127533981	0.018295012
*Bacteroides fragilis*_638R	0.01629289	0.00052618	0.03163936	0.251830001	0.013538903
*Bacteroides thetaiotaomicron*	0.009800153	0.000891472	0.020986528	0.27865706	0.016334126
*Streptococcus mutans*	3.98921E-05	0.000237374	0.001342484	0.031289194	0.074286401

**Table 4:** The dominant and statistically significant taxon at phylum and genus levels among preoperative and postoperative group of CC patients with SKYD syndrome and healthy control group.

Taxon	Mean in control	Mean in SKYD-pre	Mean in SKYD-post	FDR	P.adj
Phylum					
Proteobacteria	0.074885919	0.501887374	0.101456562	1.16046E-20	1.49041E-11
Firmicutes	0.592219166	0.392300747	0.753146375	4.88342E-08	1.6659E-09
Bacteroidetes	0.228952951	0.052028689	0.031094709	1.08835E-05	0.861733197
Actinobacteria	0.038880298	0.034275185	0.102352724	0.011241164	0.0067449
Verrucomicrobia	0.043770178	0.004204932	0.008325756	0.049267684	0.960021385
Fusobacteria	0.004387436	0.010880848	0.002157209	0.443407543	0.209676368
Genus					
*Escherichia*/*Shigella*	0.047163577	0.409221779	0.053678249	1.87842E-16	1.49103E-11
*Lachnospiracea_incertae_sedis*	0.046385493	0.03444256	0.123615791	0.000148215	6.89613E-06
*Anaerostipes*	0.002919169	0.001522526	0.018395108	0.010420084	0.001367937
*Bacteroides*	0.128307804	0.038973433	0.027029886	0.012705136	0.907514286
*Blautia*	0.058885832	0.050686752	0.141940775	0.018338293	0.002622173
*Clostridium*_XVIII	0.008219252	0.012365201	0.055020116	0.01953453	0.008206717
*Dorea*	0.013300126	0.005402985	0.035876225	0.024595064	0.001717147
*Fusicatenibacter*	0.003086289	0.001572445	0.01733136	0.030444757	0.004863631

## Data Availability

The raw data supporting the conclusions of this article will be made available by the authors without undue reservation.

## Supplementary Materials

Supplementary Materials Table S1: Detailed statistics of the sequencing data of each sample from each group. Table S2: Detailed characteristics of community diversity for each sample from each group. Table S3: Dominant and differential bacterial species among preoperative, postoperative group of CC patients and a healthy control group. Table S4: Specific and differential bacterial species among the preoperative group of CC patients with the five TCM syndromes and a healthy control group. Table S5: Specific and differential bacterial species among the postoperative group of CC patients with the five TCM syndromes and healthy control group.
