# Supplementary material for: Study of Correlation between Intestinal Microbiota and Traditional Chinese Medicine Syndrome of Patients with Colon Cancer
Source: Evid Based Complement Alternat Med. 2022 Jul 11;2022:2989456. doi: 10.1155/2022/2989456 (PMC9293549; doi:10.1155/2022/2989456)
Supplement: Supplementary Materials — Table S1: Detailed statistics of the sequencing data of each sample from each group. Table S2: Detailed characteristics of community diversity for each sample from each group. Table S3: Dominant and differential bacterial species among preoperative, postoperative group of CC patients and a healthy control group. Table S4: Specific and differential bacterial species among the preoperative group of CC patients with the five TCM syndromes and a healthy control group. Table S5: Specific and differential bacterial species among the postoperative group of CC patients with the five TCM syndromes and healthy control group. [file 2989456.f1.doc]

*Supplementary materials*

Table S1 Detailed quality control statistics of the sequencing data of each sample from each groups

| Groups | Samples | Raw reads | Q20  (%) | Q30  (%) | Merge | Merge  (%) | Primer | Clean reads | Clean reads (%) | Q20  (%) | Q30  (%) |
| --- | --- | --- | --- | --- | --- | --- | --- | --- | --- | --- | --- |
| DHA-pre | 132 | 86964 | 97.88 | 93.71 | 79485 | 91.4 | 79156 | 70436 | 80.99 | 99.01 | 96.01 |
| DHA-pre | 53 | 95721 | 97.95 | 93.92 | 88269 | 92.21 | 87952 | 77837 | 81.32 | 99.03 | 96.03 |
| DHA-pre | 174 | 98534 | 98.07 | 94.14 | 91596 | 92.96 | 91236 | 81284 | 82.49 | 99.06 | 96.05 |
| DHA-pre | 130 | 109759 | 97.86 | 93.71 | 99758 | 90.89 | 99207 | 87309 | 79.55 | 99.01 | 95.97 |
| DHA-pre | 43 | 60824 | 97.36 | 92.59 | 56154 | 92.32 | 55849 | 49022 | 80.6 | 98.95 | 95.74 |
| DHA-pre | 21 | 91226 | 97.98 | 93.94 | 83888 | 91.96 | 83366 | 74174 | 81.31 | 99.03 | 96 |
| DHA-pre | 12 | 98263 | 98.01 | 94.02 | 90955 | 92.56 | 90435 | 79135 | 80.53 | 98.95 | 95.79 |
| DHA-pre | 9 | 93387 | 97.94 | 93.88 | 85361 | 91.41 | 84574 | 75233 | 80.56 | 99.04 | 96 |
| DHA-pre | 92 | 46140 | 95.9 | 87.85 | 45403 | 98.4 | 45145 | 36749 | 79.65 | 97.85 | 91.86 |
| DHA-pre | 17 | 75991 | 97.54 | 92.87 | 68402 | 90.01 | 67918 | 57736 | 75.98 | 98.77 | 95.17 |
| DHA-pre | 134 | 108323 | 97.96 | 94.06 | 100540 | 92.82 | 100167 | 89353 | 82.49 | 99.02 | 96.01 |
| DHA-pre | 23 | 105920 | 97.95 | 93.93 | 97299 | 91.86 | 96641 | 85322 | 80.55 | 99.04 | 96.03 |
| DHA-pre | 114 | 97385 | 97.36 | 92.33 | 87868 | 90.23 | 87507 | 74789 | 76.8 | 98.8 | 95.22 |
| DHA-pre | 159 | 116606 | 98.01 | 94.06 | 105478 | 90.46 | 104642 | 92198 | 79.07 | 99.01 | 95.93 |
| DHA-pre | 20 | 75359 | 98.04 | 94.1 | 70849 | 94.02 | 70365 | 62562 | 83.02 | 98.99 | 95.92 |
| DHA-pre | 93 | 96732 | 97.64 | 93.24 | 85660 | 88.55 | 85442 | 73637 | 76.12 | 98.88 | 95.53 |
| DHA-pre | 111 | 97258 | 97.86 | 93.77 | 90019 | 92.56 | 89719 | 79156 | 81.39 | 98.97 | 95.92 |
| DHA-pre | 117 | 61968 | 97.79 | 93.64 | 56843 | 91.73 | 56544 | 49857 | 80.46 | 99 | 95.94 |
| DHA-pre | 2 | 68575 | 97.73 | 93.19 | 60911 | 88.82 | 60159 | 50863 | 74.17 | 98.75 | 95.12 |
| DHA-pre | 56 | 88283 | 97.39 | 92.36 | 80291 | 90.95 | 79826 | 66882 | 75.76 | 98.58 | 94.46 |
| DHA-pre | 37 | 108420 | 97.89 | 93.93 | 99690 | 91.95 | 98897 | 88746 | 81.85 | 99.11 | 96.29 |
| DHA-pre | 31 | 94308 | 97.96 | 93.98 | 88889 | 94.25 | 88325 | 78781 | 83.54 | 99.04 | 95.99 |
| SDD-pre | 62 | 93208 | 97.92 | 93.96 | 85175 | 91.38 | 84732 | 75527 | 81.03 | 99.11 | 96.2 |
| SDD-pre | 82 | 91144 | 97.91 | 93.89 | 84243 | 92.43 | 83981 | 74077 | 81.27 | 99.05 | 96.1 |
| SDD-pre | 131 | 92628 | 97.78 | 93.65 | 84323 | 91.03 | 83986 | 75108 | 81.09 | 99.12 | 96.32 |
| SDD-pre | 85 | 98254 | 98.07 | 94.28 | 91592 | 93.22 | 91262 | 81548 | 83 | 99.1 | 96.25 |
| SDD-pre | 18 | 73506 | 98 | 94.06 | 67542 | 91.89 | 67055 | 59946 | 81.55 | 99.01 | 95.94 |
| SDD-pre | 74 | 120280 | 97.73 | 93.45 | 109726 | 91.23 | 108997 | 96454 | 80.19 | 98.99 | 95.89 |
| SDD-pre | 189 | 101953 | 97.9 | 93.75 | 94298 | 92.49 | 94056 | 81931 | 80.36 | 98.97 | 95.79 |
| SDD-pre | 148 | 103308 | 98.01 | 93.9 | 94963 | 91.92 | 94567 | 82480 | 79.84 | 98.92 | 95.72 |
| SDD-pre | 54 | 101447 | 97.57 | 92.92 | 90096 | 88.81 | 89613 | 75947 | 74.86 | 98.77 | 95.18 |
| SDD-pre | 57 | 90943 | 97.85 | 93.66 | 84218 | 92.61 | 83817 | 74982 | 82.45 | 99.08 | 96.22 |
| SDD-pre | 147 | 84189 | 98.15 | 94.42 | 77091 | 91.57 | 76814 | 68810 | 81.73 | 99.13 | 96.33 |
| SDD-pre | 65 | 92175 | 98.04 | 94.16 | 85808 | 93.09 | 85257 | 76359 | 82.84 | 99.06 | 96.14 |
| SDD-pre | 184 | 115014 | 97.98 | 93.97 | 106117 | 92.26 | 105822 | 94093 | 81.81 | 98.98 | 95.89 |
| SDD-pre | 26 | 99415 | 97.81 | 93.59 | 91065 | 91.6 | 90810 | 81052 | 81.53 | 99.01 | 95.91 |
| SDD-pre | 187 | 94655 | 97.31 | 92.31 | 88178 | 93.16 | 87775 | 77820 | 82.21 | 98.99 | 95.8 |
| SKYD-pre | 107 | 98562 | 98.03 | 94.17 | 91374 | 92.71 | 90982 | 80837 | 82.02 | 98.97 | 95.84 |
| SKYD-pre | 28 | 56687 | 98.11 | 94.26 | 52934 | 93.38 | 52419 | 46036 | 81.21 | 98.91 | 95.67 |
| SKYD-pre | 101 | 95915 | 98.07 | 94.19 | 88480 | 92.25 | 88209 | 78433 | 81.77 | 99 | 95.89 |
| SKYD-pre | 63 | 67892 | 98.12 | 94.34 | 63849 | 94.04 | 63517 | 57238 | 84.31 | 99.11 | 96.24 |
| SKYD-pre | 125 | 84007 | 97.4 | 92.49 | 77447 | 92.19 | 77205 | 67648 | 80.53 | 98.9 | 95.49 |
| SKYD-pre | 70 | 107915 | 97.97 | 93.98 | 99482 | 92.19 | 98902 | 87736 | 81.3 | 99.04 | 96.02 |
| SKYD-pre | 81 | 76901 | 97.76 | 93.34 | 71192 | 92.58 | 70979 | 62390 | 81.13 | 98.94 | 95.66 |
| SKYD-pre | 170 | 120639 | 97.8 | 93.49 | 110922 | 91.95 | 110614 | 97666 | 80.96 | 98.97 | 95.81 |
| SKYD-pre | 73 | 102882 | 97.81 | 93.58 | 93929 | 91.3 | 93383 | 82314 | 80.01 | 98.96 | 95.74 |
| SKYD-pre | 120 | 106946 | 97.81 | 93.61 | 97769 | 91.42 | 97344 | 85670 | 80.11 | 99.02 | 95.93 |
| SKYD-pre | 102 | 83657 | 97.21 | 91.66 | 76644 | 91.62 | 76309 | 60909 | 72.81 | 98.4 | 93.83 |
| SKYD-pre | 186 | 117096 | 98.02 | 94.07 | 106671 | 91.1 | 106361 | 91383 | 78.04 | 98.94 | 95.69 |
| SKYD-pre | 154 | 103193 | 97.52 | 92.83 | 94360 | 91.44 | 94071 | 82203 | 79.66 | 98.91 | 95.58 |
| SKYD-pre | 129 | 95511 | 97.99 | 93.87 | 90230 | 94.47 | 89722 | 79708 | 83.45 | 98.89 | 95.55 |
| SKYD-pre | 30 | 85952 | 97.87 | 93.77 | 79339 | 92.31 | 78825 | 69599 | 80.97 | 99.04 | 96 |
| SKYD-pre | 39 | 45944 | 98.27 | 94.51 | 44979 | 97.9 | 44641 | 41795 | 90.97 | 99.1 | 96.08 |
| SKYD-pre | 135 | 78987 | 98.04 | 94.15 | 74011 | 93.7 | 73698 | 66091 | 83.67 | 99.02 | 96 |
| LKYD-pre | 47 | 87251 | 97.68 | 93.32 | 79890 | 91.56 | 79473 | 69501 | 79.66 | 98.96 | 95.73 |
| LKYD-pre | 192 | 108255 | 98 | 94.07 | 99917 | 92.3 | 99579 | 88962 | 82.18 | 99.08 | 96.2 |
| LKYD-pre | 181 | 98633 | 97.36 | 92.73 | 88677 | 89.91 | 88332 | 75327 | 76.37 | 98.83 | 95.33 |
| LKYD-pre | 190 | 94845 | 97.97 | 93.98 | 87870 | 92.65 | 87471 | 77485 | 81.7 | 99.02 | 95.97 |
| LKYD-pre | 165 | 101160 | 97.7 | 93.16 | 93260 | 92.19 | 92978 | 80877 | 79.95 | 98.92 | 95.62 |
| LKYD-pre | 146 | 81647 | 97.2 | 91.73 | 73687 | 90.25 | 73201 | 57299 | 70.18 | 98.24 | 93.33 |
| LKYD-pre | 163 | 89751 | 98.23 | 94.41 | 84742 | 94.42 | 84404 | 74708 | 83.24 | 98.91 | 95.62 |
| LKYD-pre | 193 | 102095 | 97.88 | 93.75 | 95991 | 94.02 | 95626 | 84470 | 82.74 | 99 | 95.97 |
| LKYD-pre | 155 | 87705 | 97.94 | 93.82 | 80394 | 91.66 | 80161 | 70255 | 80.1 | 99.02 | 95.95 |
| LKYD-pre | 157 | 85077 | 97.66 | 93.11 | 74561 | 87.64 | 74066 | 61352 | 72.11 | 98.69 | 94.94 |
| LKYD-pre | 124 | 90436 | 97.95 | 93.86 | 82185 | 90.88 | 81914 | 70693 | 78.17 | 98.99 | 95.81 |
| LKYD-pre | 164 | 99682 | 98.12 | 94.27 | 92281 | 92.58 | 92024 | 81852 | 82.11 | 98.98 | 95.87 |
| QBD-pre | 138 | 102270 | 97.78 | 93.44 | 96112 | 93.98 | 95823 | 84945 | 83.06 | 98.92 | 95.7 |
| QBD-pre | 48 | 54406 | 98.13 | 94.25 | 50374 | 92.59 | 49907 | 45043 | 82.79 | 99.15 | 96.44 |
| QBD-pre | 32 | 43662 | 98.03 | 94.2 | 40643 | 93.09 | 40281 | 36255 | 83.04 | 99.06 | 96.13 |
| QBD-pre | 142 | 121946 | 98.28 | 94.63 | 112079 | 91.91 | 111722 | 101895 | 83.56 | 99.19 | 96.56 |
| QBD-pre | 176 | 101709 | 97.63 | 93.07 | 95089 | 93.49 | 94802 | 82972 | 81.58 | 98.89 | 95.53 |
| QBD-pre | 27 | 64738 | 98.01 | 94.09 | 59924 | 92.56 | 59466 | 53332 | 82.38 | 99.04 | 96.03 |
| QBD-pre | 97 | 103595 | 98.06 | 94.12 | 95621 | 92.3 | 95350 | 85219 | 82.26 | 98.98 | 95.91 |
| QBD-pre | 6 | 66176 | 97.87 | 93.73 | 60236 | 91.02 | 59642 | 52438 | 79.24 | 98.99 | 95.85 |
| QBD-pre | 156 | 114671 | 97.99 | 94 | 106650 | 93.01 | 106379 | 95145 | 82.97 | 99.08 | 96.19 |
| QBD-pre | 96 | 77805 | 97.83 | 93.39 | 71041 | 91.31 | 70793 | 60275 | 77.47 | 98.83 | 95.24 |
| QBD-pre | 67 | 45605 | 97.83 | 93.54 | 40980 | 89.86 | 40518 | 34404 | 75.44 | 98.85 | 95.4 |
| QBD-pre | 180 | 101383 | 97.72 | 93.27 | 92114 | 90.86 | 91856 | 80196 | 79.1 | 99.02 | 95.92 |
| QBD-pre | 173 | 73817 | 98.1 | 94.26 | 68017 | 92.14 | 67627 | 59953 | 81.22 | 98.97 | 95.83 |
| QBD-pre | 78 | 97078 | 97.65 | 93.15 | 85130 | 87.69 | 84563 | 71684 | 73.84 | 98.8 | 95.3 |
| QBD-pre | 152 | 80588 | 97.84 | 93.55 | 73238 | 90.88 | 72969 | 62785 | 77.91 | 98.94 | 95.63 |
| QBD-pre | 4 | 63035 | 98.01 | 93.96 | 58739 | 93.18 | 58116 | 51060 | 81 | 98.92 | 95.65 |
| QBD-pre | 139 | 90554 | 98.03 | 94.07 | 84690 | 93.52 | 84454 | 74350 | 82.11 | 98.97 | 95.87 |
| QBD-pre | 167 | 94711 | 97.89 | 93.78 | 86963 | 91.82 | 86727 | 76243 | 80.5 | 99.01 | 95.9 |
| Control | 110 | 115701 | 97.84 | 93.65 | 107171 | 92.63 | 106836 | 95448 | 82.5 | 99.05 | 96.12 |
| Control | 166 | 124244 | 97.99 | 94.08 | 114073 | 91.81 | 113794 | 102055 | 82.14 | 99.1 | 96.28 |
| Control | 25 | 86795 | 97.86 | 93.76 | 79430 | 91.51 | 78911 | 69701 | 80.31 | 99.05 | 96.11 |
| Control | 8 | 76377 | 97.14 | 91.74 | 69568 | 91.09 | 69000 | 57928 | 75.84 | 98.61 | 94.57 |
| Control | 10 | 58002 | 97.95 | 93.88 | 54178 | 93.41 | 53684 | 48079 | 82.89 | 99.05 | 96.12 |
| Control | 94 | 76715 | 97.96 | 93.92 | 70068 | 91.34 | 69864 | 61042 | 79.57 | 98.93 | 95.77 |
| Control | 143 | 100852 | 97.69 | 93.26 | 93549 | 92.76 | 93089 | 82429 | 81.73 | 98.91 | 95.56 |
| Control | 24 | 91471 | 97.58 | 93.03 | 82894 | 90.62 | 82213 | 71348 | 78 | 98.88 | 95.45 |
| Control | 3 | 91685 | 97.94 | 93.96 | 85101 | 92.82 | 84282 | 75880 | 82.76 | 99.09 | 96.24 |
| Control | 113 | 129916 | 96.73 | 90.84 | 119789 | 92.2 | 119481 | 103492 | 79.66 | 98.81 | 95.14 |
| Control | 44 | 101868 | 97.45 | 92.56 | 91312 | 89.64 | 90750 | 77043 | 75.63 | 98.75 | 95.08 |
| Control | 1 | 81969 | 98.02 | 94.04 | 75665 | 92.31 | 74998 | 67355 | 82.17 | 99.07 | 96.15 |
| Control | 68 | 87889 | 97.8 | 93.6 | 80318 | 91.39 | 79767 | 70407 | 80.11 | 99.03 | 95.97 |
| Control | 169 | 114878 | 96.51 | 90.04 | 105096 | 91.48 | 104760 | 85416 | 74.35 | 98.36 | 93.55 |
| Control | 83 | 85729 | 97.78 | 93.38 | 80848 | 94.31 | 80589 | 71291 | 83.16 | 98.86 | 95.46 |
| Control | 38 | 96172 | 97.85 | 93.71 | 90514 | 94.12 | 89845 | 81220 | 84.45 | 99.1 | 96.21 |
| Control | 104 | 109856 | 97.13 | 91.8 | 101180 | 92.1 | 100460 | 87789 | 79.91 | 98.84 | 95.33 |
| Control | 185 | 118338 | 97.94 | 93.89 | 109086 | 92.18 | 108764 | 97407 | 82.31 | 99.11 | 96.28 |
| Control | 175 | 119377 | 97.61 | 93.07 | 110189 | 92.3 | 109764 | 97472 | 81.65 | 99 | 95.87 |
| Control | 13 | 81929 | 96.86 | 91.08 | 73550 | 89.77 | 72990 | 60485 | 73.83 | 98.56 | 94.24 |
| Control | 14 | 68242 | 97.63 | 93.13 | 60823 | 89.13 | 60253 | 51807 | 75.92 | 98.87 | 95.45 |
| Control | 15 | 114486 | 97.73 | 93.45 | 104104 | 90.93 | 103723 | 92287 | 80.61 | 99.05 | 96.07 |
| Control | 182 | 129126 | 97.74 | 93.47 | 116358 | 90.11 | 116004 | 101279 | 78.43 | 98.96 | 95.79 |
| Control | 55 | 135156 | 97.91 | 93.84 | 121209 | 89.68 | 120860 | 105741 | 78.24 | 98.99 | 95.9 |
| DHA-post | 105 | 107452 | 97.74 | 93.51 | 99700 | 92.79 | 99270 | 88010 | 81.91 | 99.03 | 95.93 |
| DHA-post | 194 | 100072 | 97.86 | 93.74 | 91926 | 91.86 | 91537 | 81303 | 81.24 | 99.04 | 96.05 |
| DHA-post | 77 | 93360 | 97.85 | 93.78 | 86073 | 92.19 | 85441 | 76037 | 81.44 | 99.03 | 95.97 |
| DHA-post | 11 | 87849 | 97.77 | 93.57 | 80651 | 91.81 | 80007 | 71199 | 81.05 | 99.04 | 96.01 |
| DHA-post | 58 | 106180 | 97.82 | 93.63 | 98563 | 92.83 | 97990 | 87379 | 82.29 | 99.03 | 96 |
| DHA-post | 86 | 102197 | 97.84 | 93.72 | 93548 | 91.54 | 93257 | 82766 | 80.99 | 99.06 | 96.08 |
| DHA-post | 19 | 77659 | 97.62 | 93.21 | 71030 | 91.46 | 70553 | 62585 | 80.59 | 99.06 | 96.06 |
| DHA-post | 149 | 120531 | 97.56 | 93.08 | 109692 | 91.01 | 109326 | 96284 | 79.88 | 99 | 95.87 |
| DHA-post | 52 | 100531 | 95.37 | 87.45 | 90803 | 90.32 | 90314 | 70483 | 70.11 | 98.16 | 92.9 |
| DHA-post | 137 | 110470 | 97.69 | 93.33 | 100125 | 90.64 | 99809 | 87492 | 79.2 | 98.93 | 95.64 |
| DHA-post | 33 | 71444 | 97.53 | 92.99 | 65566 | 91.77 | 65079 | 57507 | 80.49 | 99 | 95.85 |
| SDD-post | 69 | 84240 | 97.88 | 93.85 | 78572 | 93.27 | 78156 | 69852 | 82.92 | 99.1 | 96.22 |
| SDD-post | 161 | 100130 | 97.92 | 93.8 | 92825 | 92.7 | 92514 | 81833 | 81.73 | 98.98 | 95.87 |
| SDD-post | 177 | 97386 | 97.83 | 93.64 | 88484 | 90.86 | 88160 | 76557 | 78.61 | 98.89 | 95.63 |
| SDD-post | 158 | 109645 | 95.12 | 86.74 | 98962 | 90.26 | 98398 | 71859 | 65.54 | 97.91 | 92.08 |
| SDD-post | 59 | 100136 | 97.91 | 93.73 | 92625 | 92.5 | 92059 | 80964 | 80.85 | 98.96 | 95.79 |
| SDD-post | 91 | 90841 | 97.85 | 93.72 | 84112 | 92.59 | 83852 | 75083 | 82.65 | 99.09 | 96.21 |
| SDD-post | 119 | 57586 | 98.16 | 94.39 | 52996 | 92.03 | 52712 | 47196 | 81.96 | 99.12 | 96.38 |
| SDD-post | 41 | 60440 | 97.88 | 93.7 | 55393 | 91.65 | 55059 | 47430 | 78.47 | 98.82 | 95.42 |
| SDD-post | 49 | 103579 | 98.06 | 94.18 | 97330 | 93.97 | 96863 | 86395 | 83.41 | 99.07 | 96.16 |
| SDD-post | 191 | 90300 | 97.46 | 92.7 | 82141 | 90.96 | 81701 | 71747 | 79.45 | 98.96 | 95.77 |
| SDD-post | 5 | 84035 | 97.87 | 93.72 | 77584 | 92.32 | 77017 | 69001 | 82.11 | 99.04 | 96.09 |
| SDD-post | 98 | 116850 | 97.27 | 92.24 | 107877 | 92.32 | 107620 | 95111 | 81.4 | 98.97 | 95.7 |
| SDD-post | 34 | 84568 | 98.1 | 94.28 | 80724 | 95.45 | 80146 | 72392 | 85.6 | 99.11 | 96.28 |
| SDD-post | 144 | 90437 | 98.01 | 94.07 | 83681 | 92.53 | 83123 | 75121 | 83.06 | 99.1 | 96.26 |
| SDD-post | 150 | 61644 | 97.62 | 93.13 | 55389 | 89.85 | 55199 | 47122 | 76.44 | 98.78 | 95.17 |
| SDD-post | 140 | 102822 | 97.58 | 93.12 | 91428 | 88.92 | 91009 | 76900 | 74.79 | 98.78 | 95.22 |
| SDD-post | 118 | 100897 | 98.01 | 94.05 | 93495 | 92.66 | 93111 | 82855 | 82.12 | 99.03 | 96.04 |
| SDD-post | 80 | 93383 | 98.14 | 94.44 | 87536 | 93.74 | 86129 | 77622 | 83.12 | 99.11 | 96.3 |
| SDD-post | 103 | 66269 | 98.05 | 94.2 | 62186 | 93.84 | 62005 | 55451 | 83.68 | 99.05 | 96.15 |
| SDD-post | 100 | 124595 | 97.8 | 93.57 | 113978 | 91.48 | 113679 | 99638 | 79.97 | 98.95 | 95.78 |
| SDD-post | 178 | 81032 | 97.55 | 92.89 | 74755 | 92.25 | 74487 | 65861 | 81.28 | 98.99 | 95.85 |
| SDD-post | 162 | 113071 | 97.5 | 92.76 | 100643 | 89.01 | 100180 | 83725 | 74.05 | 98.68 | 94.96 |
| SDD-post | 46 | 81402 | 97.89 | 93.7 | 73540 | 90.34 | 73129 | 63459 | 77.96 | 98.86 | 95.49 |
| SKYD-post | 45 | 102080 | 97.92 | 93.85 | 91428 | 89.57 | 90898 | 75765 | 74.22 | 98.6 | 94.82 |
| SKYD-post | 61 | 101254 | 97.41 | 92.58 | 88771 | 87.67 | 88237 | 71091 | 70.21 | 98.49 | 94.32 |
| SKYD-post | 179 | 107587 | 98.08 | 94.25 | 102644 | 95.41 | 102319 | 93136 | 86.57 | 99.1 | 96.27 |
| SKYD-post | 109 | 103043 | 98.04 | 94.18 | 95981 | 93.15 | 95677 | 83716 | 81.24 | 98.92 | 95.78 |
| SKYD-post | 36 | 104033 | 97.5 | 92.78 | 95831 | 92.12 | 95546 | 83473 | 80.24 | 98.89 | 95.47 |
| SKYD-post | 51 | 40565 | 97.94 | 93.87 | 37716 | 92.98 | 37347 | 33247 | 81.96 | 99.05 | 96.17 |
| SKYD-post | 168 | 116441 | 98.01 | 94.05 | 108461 | 93.15 | 108186 | 96553 | 82.92 | 99.02 | 96.05 |
| SKYD-post | 60 | 75049 | 98.03 | 93.9 | 71247 | 94.93 | 70811 | 63951 | 85.21 | 98.96 | 95.83 |
| SKYD-post | 16 | 79069 | 95.98 | 88.82 | 74417 | 94.12 | 73835 | 63503 | 80.31 | 98.59 | 94.39 |
| SKYD-post | 99 | 79924 | 97.97 | 94.03 | 73930 | 92.5 | 73740 | 65538 | 82 | 98.97 | 95.9 |
| SKYD-post | 75 | 99860 | 98.07 | 94.21 | 94030 | 94.16 | 93452 | 83676 | 83.79 | 99.08 | 96.2 |
| SKYD-post | 29 | 76345 | 98.1 | 94.29 | 71887 | 94.16 | 71384 | 64336 | 84.27 | 99.12 | 96.32 |
| SKYD-post | 89 | 106845 | 97.81 | 93.5 | 99028 | 92.68 | 98629 | 87076 | 81.5 | 98.97 | 95.82 |
| SKYD-post | 136 | 81091 | 97.77 | 93.5 | 74763 | 92.2 | 74539 | 65179 | 80.38 | 98.91 | 95.69 |
| SKYD-post | 72 | 87814 | 97.94 | 93.94 | 82465 | 93.91 | 81913 | 73726 | 83.96 | 99.03 | 96.04 |
| SKYD-post | 122 | 78571 | 98.14 | 94.4 | 75083 | 95.56 | 74866 | 68705 | 87.44 | 99.11 | 96.32 |
| SKYD-post | 115 | 98531 | 97.8 | 93.5 | 90794 | 92.15 | 90420 | 78640 | 79.81 | 98.93 | 95.67 |
| SKYD-post | 151 | 71228 | 98.08 | 94.27 | 66375 | 93.19 | 66164 | 59504 | 83.54 | 99.08 | 96.23 |
| SKYD-post | 123 | 99801 | 98.17 | 94.44 | 94235 | 94.42 | 93957 | 84351 | 84.52 | 99.02 | 96.04 |
| SKYD-post | 126 | 114353 | 97.91 | 93.85 | 104256 | 91.17 | 103917 | 90982 | 79.56 | 98.92 | 95.78 |
| SKYD-post | 128 | 119426 | 98.1 | 94.31 | 112245 | 93.99 | 111903 | 100232 | 83.93 | 99.06 | 96.17 |
| LKYD-post | 64 | 94509 | 97.69 | 93.4 | 86980 | 92.03 | 86376 | 77241 | 81.73 | 99.09 | 96.14 |
| LKYD-post | 108 | 118745 | 97.83 | 93.72 | 108263 | 91.17 | 107899 | 96969 | 81.66 | 99.11 | 96.24 |
| LKYD-post | 153 | 121711 | 97.82 | 93.65 | 108781 | 89.38 | 108391 | 90294 | 74.19 | 98.87 | 95.46 |
| LKYD-post | 116 | 120868 | 97.96 | 93.97 | 109890 | 90.92 | 109568 | 96333 | 79.7 | 98.96 | 95.75 |
| LKYD-post | 127 | 134119 | 97.72 | 93.51 | 121977 | 90.95 | 121562 | 108167 | 80.65 | 99.08 | 96.1 |
| LKYD-post | 160 | 107441 | 97.84 | 93.66 | 98171 | 91.37 | 97795 | 87042 | 81.01 | 99.02 | 95.94 |
| LKYD-post | 42 | 86409 | 97.69 | 93.27 | 79194 | 91.65 | 78780 | 70628 | 81.74 | 99.03 | 95.92 |
| LKYD-post | 7 | 60136 | 97.58 | 93.11 | 54645 | 90.87 | 54196 | 47216 | 78.52 | 99 | 95.79 |
| LKYD-post | 133 | 94643 | 97.43 | 92.7 | 85715 | 90.57 | 85266 | 74663 | 78.89 | 98.96 | 95.7 |
| LKYD-post | 66 | 87639 | 97.65 | 93.28 | 80818 | 92.22 | 80315 | 71206 | 81.25 | 99.02 | 95.89 |
| LKYD-post | 22 | 90836 | 97.75 | 93.52 | 82521 | 90.85 | 81949 | 72711 | 80.05 | 99.03 | 95.92 |
| QBD-post | 95 | 101580 | 97.82 | 93.61 | 92805 | 91.36 | 92596 | 81481 | 80.21 | 98.96 | 95.8 |
| QBD-post | 87 | 89757 | 97.71 | 93.29 | 80816 | 90.04 | 80503 | 67645 | 75.36 | 98.76 | 95.12 |
| QBD-post | 40 | 65201 | 97.9 | 93.78 | 59899 | 91.87 | 58026 | 50652 | 77.69 | 99 | 95.86 |
| QBD-post | 76 | 100288 | 97.96 | 94.02 | 91727 | 91.46 | 91170 | 81186 | 80.95 | 99.03 | 96.04 |
| QBD-post | 172 | 113771 | 98.09 | 94.24 | 105020 | 92.31 | 104734 | 93445 | 82.13 | 99.01 | 95.93 |
| QBD-post | 183 | 104865 | 97.5 | 92.7 | 94452 | 90.07 | 94156 | 82402 | 78.58 | 98.87 | 95.38 |
| QBD-post | 171 | 117490 | 97.84 | 93.69 | 107899 | 91.84 | 107613 | 95720 | 81.47 | 99.02 | 95.95 |
| QBD-post | 50 | 39763 | 96.91 | 91.4 | 35476 | 89.22 | 35054 | 28875 | 72.62 | 98.47 | 94.09 |
| QBD-post | 35 | 88618 | 98.01 | 94.11 | 82829 | 93.47 | 82192 | 73678 | 83.14 | 99.06 | 96.09 |
| QBD-post | 90 | 83884 | 97.79 | 93.59 | 76850 | 91.61 | 76619 | 67742 | 80.76 | 99.02 | 95.95 |
| QBD-post | 141 | 94028 | 98.11 | 94.26 | 87016 | 92.54 | 86763 | 77886 | 82.83 | 99.04 | 96.01 |
| QBD-post | 84 | 103048 | 96.83 | 91.07 | 92368 | 89.64 | 91916 | 68270 | 66.25 | 98.02 | 92.81 |
| QBD-post | 188 | 120821 | 97.85 | 93.67 | 111271 | 92.1 | 110928 | 98374 | 81.42 | 99.01 | 95.95 |
| QBD-post | 112 | 97380 | 96.13 | 89.28 | 89286 | 91.69 | 88973 | 75936 | 77.98 | 98.55 | 94.3 |
| QBD-post | 71 | 108034 | 97.85 | 93.65 | 98292 | 90.98 | 97753 | 85775 | 79.4 | 98.93 | 95.65 |
| QBD-post | 121 | 108494 | 97.89 | 93.76 | 100218 | 92.37 | 99959 | 87518 | 80.67 | 98.98 | 95.76 |
| QBD-post | 88 | 108162 | 97.85 | 93.67 | 98425 | 91 | 98104 | 87159 | 80.58 | 99.05 | 95.99 |
| QBD-post | 106 | 91300 | 98.05 | 93.98 | 81712 | 89.5 | 81384 | 70920 | 77.68 | 98.93 | 95.6 |

Sample: sample number; Raw reads: original sequence number; Q20(%): The percentage of bases with a quality greater than 20 in the original data; Q30(%): The proportion of bases with a quality greater than 30 in the original data; Merge: the number of sequences after splicing; Merge(%): The percentage of the spliced sequence in the original sequence; Primer: the number of sequences after primers are found and removed; Clean reads: the final optimized sequence; Clean reads(%): The percentage of the optimized sequence to the original sequence; Q20(%): The proportion of bases with a quality greater than 20 in the optimized sequence; Q30(%): The proportion of bases with a quality greater than 30 in the optimized sequence.

Table S2 Detailed characteristics of community diversity for each sample(Sample: sample number; Index of community richness contain Observed(number of OTU directly observed), Chao1(used to estimate the total number of species in the sample, the larger the value, the more species) and ACE(used to estimate the total number of species in the sample, the larger the value, the more species); Index of Community diversity contain Shannon(used to estimate the diversity index of microorganisms in the sample. The larger the Shannon value, the higher the community diversity), Simpson(used to estimate the diversity index of microorganisms in the sample. The larger the Simpson index value, the lower the community diversity) and Coverage(refers to the coverage rate of each sample library. The higher the value, the higher the probability that the sequence in the sample will be detected, and the lower the probability of not being detected). )

| Groups | Sample | Observed | Chao1 | ACE | Shannon | Simpson | Coverage |
| --- | --- | --- | --- | --- | --- | --- | --- |
| DHA-pre | 132 | 270 | 439.75 | 405.3034 | 3.2735501 | 0.06929471 | 0.9971568 |
| DHA-pre | 53 | 195 | 343.5714 | 289.2455 | 3.0562865 | 0.08535699 | 0.9981142 |
| DHA-pre | 174 | 225 | 354.1111 | 333.2877 | 1.6377493 | 0.40211745 | 0.997563 |
| DHA-pre | 130 | 411 | 607.2656 | 640.9866 | 2.8935134 | 0.13084569 | 0.9953871 |
| DHA-pre | 43 | 263 | 384.5385 | 369.4208 | 3.4355825 | 0.05950741 | 0.997679 |
| DHA-pre | 21 | 249 | 320 | 339.894 | 3.3435392 | 0.06804127 | 0.9979402 |
| DHA-pre | 12 | 284 | 367.2174 | 387.4927 | 3.3357268 | 0.0723708 | 0.997447 |
| DHA-pre | 9 | 194 | 234.1351 | 261.2739 | 2.2111736 | 0.30707973 | 0.9984044 |
| DHA-pre | 92 | 298 | 405.25 | 355.7694 | 3.454565 | 0.07302324 | 0.9980852 |
| DHA-pre | 17 | 222 | 324.24 | 321.7229 | 3.0567875 | 0.09017151 | 0.9979111 |
| DHA-pre | 134 | 192 | 258.5 | 272.2977 | 3.0923938 | 0.08310605 | 0.9983463 |
| DHA-pre | 23 | 205 | 307.7 | 320.0185 | 3.0942395 | 0.07280033 | 0.9977081 |
| DHA-pre | 114 | 312 | 431.3846 | 455.2145 | 3.2920212 | 0.11449611 | 0.9971859 |
| DHA-pre | 159 | 180 | 364.25 | 299.3865 | 2.757316 | 0.13503744 | 0.9980562 |
| DHA-pre | 20 | 158 | 243.55 | 241.1021 | 2.1100997 | 0.20785976 | 0.9982883 |
| DHA-pre | 93 | 292 | 358.2791 | 368.8988 | 3.0839895 | 0.12176558 | 0.9977951 |
| DHA-pre | 111 | 218 | 305 | 289.6383 | 3.1127565 | 0.09866387 | 0.9983173 |
| DHA-pre | 117 | 196 | 278.5 | 287.1272 | 2.9264168 | 0.09079155 | 0.9980852 |
| DHA-pre | 2 | 242 | 321.2273 | 340.6981 | 2.1250185 | 0.26059458 | 0.997563 |
| DHA-pre | 56 | 243 | 302.2941 | 317.9362 | 2.6721189 | 0.19167708 | 0.9981433 |
| DHA-pre | 37 | 201 | 309.04 | 312.1621 | 2.3507453 | 0.1993358 | 0.9978531 |
| DHA-pre | 31 | 192 | 287 | 321.9217 | 1.7929965 | 0.38015424 | 0.9977951 |
| SDD-pre | 62 | 92 | 146.0909 | 156.7378 | 2.1328401 | 0.17038323 | 0.9989846 |
| SDD-pre | 82 | 189 | 245 | 250.8142 | 3.09916 | 0.08634938 | 0.9985784 |
| SDD-pre | 131 | 283 | 410.6 | 394.2358 | 3.1382308 | 0.14972914 | 0.997447 |
| SDD-pre | 85 | 150 | 202.1053 | 212.275 | 1.945669 | 0.3409464 | 0.9986945 |
| SDD-pre | 18 | 217 | 329.8947 | 306.177 | 3.1513322 | 0.07654574 | 0.9980852 |
| SDD-pre | 74 | 174 | 269.4 | 246.1224 | 2.8894001 | 0.1021598 | 0.9984334 |
| SDD-pre | 189 | 276 | 351.303 | 351.3257 | 3.3142887 | 0.09440458 | 0.9979402 |
| SDD-pre | 148 | 178 | 234 | 231.8723 | 2.3620207 | 0.17732844 | 0.9985784 |
| SDD-pre | 54 | 175 | 284.4737 | 284.8142 | 2.4126733 | 0.1544011 | 0.9981142 |
| SDD-pre | 57 | 158 | 245.3529 | 235.1454 | 2.5857561 | 0.12024134 | 0.9984043 |
| SDD-pre | 147 | 219 | 289.3846 | 283.1869 | 2.2731388 | 0.20631665 | 0.9982303 |
| SDD-pre | 65 | 321 | 418.5 | 421.6492 | 3.1451963 | 0.11368912 | 0.9973599 |
| SDD-pre | 184 | 202 | 244 | 248.7794 | 2.8461525 | 0.11259468 | 0.9985784 |
| SDD-pre | 26 | 173 | 208.875 | 219.3252 | 3.0885167 | 0.07559489 | 0.9987815 |
| SDD-pre | 187 | 222 | 322.6364 | 339.8016 | 2.3678851 | 0.21925748 | 0.997621 |
| SKYD-pre | 107 | 137 | 203.3529 | 197.9343 | 2.3822328 | 0.15704715 | 0.9986074 |
| SKYD-pre | 28 | 225 | 342.9032 | 359.4793 | 2.3288918 | 0.23418778 | 0.997505 |
| SKYD-pre | 101 | 187 | 244.9545 | 240.6283 | 2.0067625 | 0.36929791 | 0.9985204 |
| SKYD-pre | 63 | 233 | 433.2 | 357.89 | 3.1504911 | 0.09811915 | 0.9977371 |
| SKYD-pre | 125 | 215 | 294.5652 | 287.185 | 2.8601637 | 0.12392813 | 0.9982303 |
| SKYD-pre | 70 | 172 | 233.875 | 259.6504 | 2.5500575 | 0.16084557 | 0.9984044 |
| SKYD-pre | 81 | 165 | 244.5652 | 265.4794 | 2.6409731 | 0.12110949 | 0.9982303 |
| SKYD-pre | 170 | 199 | 307.5 | 269.4614 | 2.2377704 | 0.25437443 | 0.9981722 |
| SKYD-pre | 73 | 120 | 186.3529 | 223.6024 | 1.8354643 | 0.35747331 | 0.9986074 |
| SKYD-pre | 120 | 195 | 348.2143 | 302.9684 | 2.5775188 | 0.16274634 | 0.9980852 |
| SKYD-pre | 102 | 162 | 235.75 | 237.7646 | 1.6161148 | 0.3471811 | 0.9982593 |
| SKYD-pre | 186 | 187 | 242.25 | 245.5503 | 2.7271789 | 0.14684008 | 0.9984914 |
| SKYD-pre | 154 | 139 | 184.8824 | 194.4548 | 2.437701 | 0.17048122 | 0.9988395 |
| SKYD-pre | 129 | 248 | 358.7 | 348.3633 | 2.1377553 | 0.30044326 | 0.997621 |
| SKYD-pre | 30 | 208 | 428.0588 | 355.2101 | 2.0594312 | 0.32028083 | 0.9974759 |
| SKYD-pre | 39 | 202 | 293.9355 | 326.3259 | 1.9651395 | 0.35944658 | 0.9977951 |
| SKYD-pre | 135 | 136 | 181.15 | 190.7192 | 2.1646496 | 0.1956467 | 0.9987525 |
| LKYD-pre | 47 | 140 | 239.5263 | 271.1785 | 2.258749 | 0.1406995 | 0.9982013 |
| LKYD-pre | 192 | 126 | 190.5 | 197.126 | 2.1337882 | 0.20620005 | 0.9987525 |
| LKYD-pre | 181 | 120 | 194.5455 | 194.8886 | 2.5121397 | 0.15157542 | 0.9988105 |
| LKYD-pre | 190 | 191 | 244.3226 | 253.692 | 2.4695332 | 0.20778992 | 0.9983173 |
| LKYD-pre | 165 | 142 | 189.5714 | 189.4497 | 2.3069331 | 0.24621319 | 0.9989266 |
| LKYD-pre | 146 | 158 | 223.8077 | 249.1011 | 2.2072054 | 0.20953984 | 0.9982883 |
| LKYD-pre | 163 | 135 | 207.0588 | 208.4271 | 1.6579652 | 0.31310876 | 0.9985494 |
| LKYD-pre | 193 | 128 | 192.4737 | 200.7249 | 2.2608416 | 0.14804816 | 0.9985494 |
| LKYD-pre | 155 | 140 | 179.2609 | 187.7555 | 1.1579483 | 0.54589666 | 0.9987525 |
| LKYD-pre | 157 | 104 | 231.5 | 192.2761 | 1.6559926 | 0.33902993 | 0.9985204 |
| LKYD-pre | 124 | 83 | 146.0769 | 179.6205 | 0.8436857 | 0.667825 | 0.9988105 |
| LKYD-pre | 164 | 109 | 157.4615 | 158.8416 | 2.2415718 | 0.17591467 | 0.9989556 |
| QBD-pre | 138 | 175 | 212.2727 | 219.0706 | 2.98879 | 0.08866767 | 0.9988105 |
| QBD-pre | 48 | 176 | 292.9062 | 302.1714 | 1.4915065 | 0.39180093 | 0.997476 |
| QBD-pre | 32 | 174 | 230 | 238.9204 | 3.2027707 | 0.06311649 | 0.9985784 |
| QBD-pre | 142 | 149 | 220.55 | 220.7475 | 1.932932 | 0.31078027 | 0.9984334 |
| QBD-pre | 176 | 184 | 227.05 | 229.0512 | 3.0242841 | 0.11186487 | 0.9987815 |
| QBD-pre | 27 | 158 | 276.3333 | 287.0412 | 1.72039 | 0.29867236 | 0.9979402 |
| QBD-pre | 97 | 184 | 272.5 | 266.9402 | 2.7865936 | 0.10854709 | 0.9982593 |
| QBD-pre | 6 | 186 | 288.7778 | 283.0073 | 1.9559357 | 0.30679201 | 0.9978241 |
| QBD-pre | 156 | 164 | 234.5 | 224.4746 | 3.2374423 | 0.05809091 | 0.9986074 |
| QBD-pre | 96 | 113 | 212.0435 | 254.8981 | 0.6339831 | 0.62274762 | 0.9980272 |
| QBD-pre | 67 | 199 | 272.7 | 269.802 | 1.3525452 | 0.51654349 | 0.9980562 |
| QBD-pre | 180 | 92 | 144.5556 | 173.3339 | 1.4599703 | 0.3676398 | 0.9987235 |
| QBD-pre | 173 | 153 | 250.5 | 254.4469 | 1.5219593 | 0.35672514 | 0.9980852 |
| QBD-pre | 78 | 157 | 229.5455 | 248.4305 | 2.6081446 | 0.10887098 | 0.9983463 |
| QBD-pre | 152 | 116 | 174.2353 | 170.2984 | 0.5128247 | 0.84125584 | 0.9986945 |
| QBD-pre | 4 | 179 | 269.0333 | 272.0989 | 1.5051649 | 0.4357208 | 0.9978531 |
| QBD-pre | 139 | 143 | 224.4762 | 242.7713 | 1.5801324 | 0.33247731 | 0.9982883 |
| QBD-pre | 167 | 171 | 304.9565 | 333.5069 | 2.0578082 | 0.23495086 | 0.9977081 |
| Control | 110 | 339 | 443.4286 | 444.2523 | 4.0472894 | 0.02991953 | 0.997505 |
| Control | 166 | 286 | 331 | 331.8632 | 3.7594782 | 0.0461989 | 0.9984044 |
| Control | 25 | 374 | 565.2857 | 492.7847 | 3.6587738 | 0.06782541 | 0.9969828 |
| Control | 8 | 401 | 582.8409 | 576.3358 | 4.0985228 | 0.03433505 | 0.9963155 |
| Control | 10 | 355 | 477.9362 | 486.0716 | 3.4970961 | 0.06500643 | 0.9968667 |
| Control | 94 | 283 | 360 | 380.0272 | 3.735265 | 0.04189452 | 0.9977661 |
| Control | 143 | 375 | 480.8182 | 483.7996 | 4.1097924 | 0.02898915 | 0.9971859 |
| Control | 24 | 296 | 378.4516 | 377.76 | 3.4514537 | 0.08101225 | 0.9979112 |
| Control | 3 | 398 | 543.3571 | 521.5087 | 3.8781407 | 0.03956612 | 0.9967797 |
| Control | 113 | 390 | 508.8333 | 491.7419 | 4.1359184 | 0.03743174 | 0.9973019 |
| Control | 44 | 328 | 452.7 | 433.0808 | 3.703099 | 0.06406397 | 0.997476 |
| Control | 1 | 394 | 507.1 | 527.3892 | 3.2640725 | 0.11779351 | 0.9966056 |
| Control | 68 | 267 | 314.5278 | 318.3912 | 3.2996337 | 0.08496001 | 0.9982883 |
| Control | 169 | 235 | 298.84 | 295.1887 | 3.5725499 | 0.05405754 | 0.9983463 |
| Control | 83 | 352 | 488.12 | 435.3201 | 3.8816764 | 0.04291791 | 0.997592 |
| Control | 38 | 378 | 527.875 | 524.3205 | 4.0633212 | 0.03773306 | 0.9968087 |
| Control | 104 | 373 | 454.0789 | 446.066 | 4.2856066 | 0.02599571 | 0.9977081 |
| Control | 185 | 288 | 355.8571 | 368.578 | 3.2567671 | 0.09082831 | 0.9977951 |
| Control | 175 | 338 | 415 | 418.6481 | 4.1062172 | 0.03213358 | 0.9977661 |
| Control | 13 | 401 | 553.6304 | 532.5474 | 3.5871013 | 0.05717327 | 0.9965476 |
| Control | 14 | 337 | 418.0789 | 413.5344 | 3.7170374 | 0.05214312 | 0.9977081 |
| Control | 15 | 377 | 494.5 | 475.8912 | 3.9393247 | 0.04483417 | 0.9972439 |
| Control | 182 | 314 | 368.2381 | 384.4435 | 3.8346444 | 0.04724447 | 0.9980272 |
| Control | 55 | 261 | 345.2857 | 320.1194 | 3.8628514 | 0.03666722 | 0.9982593 |
| DHA-post | 105 | 153 | 226.6667 | 237.1993 | 3.0485052 | 0.06893296 | 0.9984914 |
| DHA-post | 194 | 270 | 346.25 | 331.0373 | 3.7162697 | 0.05353504 | 0.9982303 |
| DHA-post | 77 | 192 | 256.6875 | 242.7317 | 3.3970915 | 0.07515299 | 0.9986655 |
| DHA-post | 11 | 288 | 382.8857 | 400.8804 | 3.6494031 | 0.05208454 | 0.997621 |
| DHA-post | 58 | 300 | 380.5 | 378.3432 | 4.0086606 | 0.03021857 | 0.9979692 |
| DHA-post | 86 | 266 | 329.2143 | 332.4661 | 3.9869465 | 0.03368566 | 0.9982593 |
| DHA-post | 19 | 307 | 417.6757 | 421.1796 | 3.5320316 | 0.06289215 | 0.9973599 |
| DHA-post | 149 | 183 | 270.3529 | 264.3612 | 2.9130971 | 0.12007394 | 0.9984043 |
| DHA-post | 52 | 337 | 441.3415 | 440.3882 | 3.6931719 | 0.0499495 | 0.9973019 |
| DHA-post | 137 | 263 | 326.5769 | 318.7138 | 3.5556313 | 0.05742561 | 0.9983173 |
| DHA-post | 33 | 282 | 489.2727 | 411.9893 | 3.532005 | 0.05367461 | 0.9972148 |
| SDD-post | 69 | 289 | 333.3333 | 339.4365 | 3.8470311 | 0.03931761 | 0.9983464 |
| SDD-post | 161 | 320 | 408.0286 | 415.0842 | 3.8267001 | 0.05915829 | 0.9977081 |
| SDD-post | 177 | 313 | 368.9149 | 385.3473 | 3.5428049 | 0.06367107 | 0.9978822 |
| SDD-post | 158 | 209 | 271.6364 | 266.0086 | 3.2625176 | 0.07069719 | 0.9984624 |
| SDD-post | 59 | 253 | 338.8 | 327.8876 | 3.5958415 | 0.04678083 | 0.9980852 |
| SDD-post | 91 | 303 | 415.4516 | 406.2039 | 3.6073624 | 0.06644674 | 0.997563 |
| SDD-post | 119 | 286 | 353.6829 | 374.9431 | 3.4711863 | 0.05299903 | 0.9978241 |
| SDD-post | 41 | 333 | 428.2326 | 431.0296 | 3.9925951 | 0.03129353 | 0.9973599 |
| SDD-post | 49 | 182 | 247.3333 | 248.6567 | 3.3017478 | 0.06875113 | 0.9985784 |
| SDD-post | 191 | 294 | 432 | 426.9819 | 3.4700381 | 0.06745573 | 0.9973019 |
| SDD-post | 5 | 325 | 402.766 | 427.1465 | 3.9260856 | 0.03388983 | 0.997505 |
| SDD-post | 98 | 308 | 363.0345 | 355.7944 | 3.8523173 | 0.048449 | 0.9983463 |
| SDD-post | 34 | 263 | 344.3571 | 333.5016 | 3.5950674 | 0.04579137 | 0.9980272 |
| SDD-post | 144 | 341 | 488.5172 | 437.6435 | 3.9462646 | 0.03428023 | 0.9973019 |
| SDD-post | 150 | 209 | 282.1818 | 310.1407 | 2.943615 | 0.09595886 | 0.9979692 |
| SDD-post | 140 | 202 | 253 | 255.8125 | 2.6795039 | 0.16535073 | 0.9985204 |
| SDD-post | 118 | 279 | 347.25 | 368.8096 | 3.6541985 | 0.04359342 | 0.9977371 |
| SDD-post | 80 | 243 | 298.4545 | 315.2077 | 3.0545504 | 0.14161163 | 0.9982303 |
| SDD-post | 103 | 264 | 312.125 | 319.9115 | 3.8021234 | 0.03629911 | 0.9983754 |
| SDD-post | 100 | 224 | 286.2174 | 283.0848 | 3.4866748 | 0.06031989 | 0.9984334 |
| SDD-post | 178 | 202 | 275.3333 | 284.513 | 2.9785709 | 0.11181641 | 0.9983753 |
| SDD-post | 162 | 230 | 368.0556 | 326.2565 | 3.6598904 | 0.03905191 | 0.9979401 |
| SDD-post | 46 | 180 | 231.1071 | 255.6919 | 3.0330606 | 0.08640881 | 0.9984334 |
| SKYD-post | 45 | 255 | 328.1818 | 336.0744 | 3.3562616 | 0.06137778 | 0.9979692 |
| SKYD-post | 61 | 213 | 333.12 | 352.8181 | 3.0303594 | 0.08821389 | 0.9977371 |
| SKYD-post | 179 | 139 | 226.5 | 230.0976 | 2.5384881 | 0.1431655 | 0.9985494 |
| SKYD-post | 109 | 185 | 313.375 | 340.2061 | 2.9436476 | 0.08875771 | 0.9977081 |
| SKYD-post | 36 | 157 | 188.3158 | 192.0853 | 3.1496961 | 0.0849099 | 0.9989846 |
| SKYD-post | 51 | 266 | 362.4865 | 379.4693 | 2.9844511 | 0.11210718 | 0.997534 |
| SKYD-post | 168 | 174 | 249.1154 | 275.9198 | 3.0816805 | 0.06829903 | 0.9981723 |
| SKYD-post | 60 | 206 | 326.6774 | 349.9649 | 2.6946415 | 0.12552597 | 0.997476 |
| SKYD-post | 16 | 213 | 322.7727 | 329.4498 | 3.0638943 | 0.08463247 | 0.9979692 |
| SKYD-post | 99 | 182 | 314.1429 | 312.5489 | 2.5425848 | 0.15434391 | 0.9978241 |
| SKYD-post | 75 | 152 | 234.65 | 247.0427 | 2.7066862 | 0.11632509 | 0.9983173 |
| SKYD-post | 29 | 189 | 289.037 | 313.817 | 2.9487341 | 0.09508005 | 0.9978531 |
| SKYD-post | 89 | 169 | 246.7727 | 247.2331 | 3.075721 | 0.06265787 | 0.9982883 |
| SKYD-post | 136 | 253 | 419.875 | 374.2404 | 2.7963595 | 0.14902427 | 0.9973889 |
| SKYD-post | 72 | 252 | 334.65 | 317.2643 | 3.6335273 | 0.04871308 | 0.9983173 |
| SKYD-post | 122 | 210 | 299.375 | 291.4912 | 2.8030694 | 0.11453455 | 0.9980852 |
| SKYD-post | 115 | 181 | 228.1429 | 233.9158 | 2.9549615 | 0.11319074 | 0.9986945 |
| SKYD-post | 151 | 266 | 457.8966 | 429.8552 | 2.1364818 | 0.27002266 | 0.9969247 |
| SKYD-post | 123 | 251 | 334.2759 | 328.7038 | 3.1549623 | 0.07748157 | 0.9979692 |
| SKYD-post | 126 | 215 | 318.8846 | 311.9053 | 3.0729899 | 0.08074202 | 0.9978531 |
| SKYD-post | 128 | 214 | 332.3333 | 314.7373 | 3.212529 | 0.06609025 | 0.9979402 |
| LKYD-post | 64 | 288 | 442.2857 | 382.8175 | 3.3776603 | 0.07619066 | 0.99765 |
| LKYD-post | 108 | 299 | 382.2759 | 365.135 | 3.5155789 | 0.0740076 | 0.9979692 |
| LKYD-post | 153 | 183 | 254.2917 | 273.2443 | 3.0226529 | 0.08078693 | 0.9982883 |
| LKYD-post | 116 | 117 | 210.5 | 168.2185 | 2.2970876 | 0.22193403 | 0.9990136 |
| LKYD-post | 127 | 249 | 340.12 | 326.3946 | 3.5283925 | 0.05306806 | 0.9980272 |
| LKYD-post | 160 | 168 | 266 | 248.3167 | 2.8767811 | 0.12383538 | 0.9985784 |
| LKYD-post | 42 | 160 | 262.2143 | 241.6335 | 3.0722876 | 0.08710631 | 0.9984334 |
| LKYD-post | 7 | 215 | 332.04 | 312.4591 | 2.5051288 | 0.16355773 | 0.9977661 |
| LKYD-post | 133 | 284 | 347.0882 | 345.9461 | 3.7090039 | 0.04400438 | 0.9980852 |
| LKYD-post | 66 | 110 | 169.5 | 157.9071 | 2.4147209 | 0.1713307 | 0.9989846 |
| LKYD-post | 22 | 252 | 360.3704 | 355.644 | 3.2959934 | 0.07919352 | 0.9977661 |
| QBD-post | 95 | 247 | 340.84 | 316.685 | 2.8525953 | 0.1309172 | 0.9979982 |
| QBD-post | 87 | 171 | 261.2308 | 277.3324 | 1.8448575 | 0.34082899 | 0.9979982 |
| QBD-post | 40 | 219 | 369.1935 | 379.0485 | 1.17828 | 0.59583198 | 0.9971858 |
| QBD-post | 76 | 195 | 278.1818 | 273.0177 | 3.0184079 | 0.07974236 | 0.9982303 |
| QBD-post | 172 | 177 | 248.55 | 268.514 | 3.1455463 | 0.07776752 | 0.9984334 |
| QBD-post | 183 | 109 | 202.5 | 150.4919 | 2.3838304 | 0.14760601 | 0.9990136 |
| QBD-post | 171 | 160 | 188.3333 | 198.0239 | 2.8179766 | 0.1343373 | 0.9989846 |
| QBD-post | 50 | 183 | 348 | 396.5165 | 1.1328048 | 0.61567137 | 0.9970988 |
| QBD-post | 35 | 211 | 341.3333 | 319.5189 | 3.2030688 | 0.09955444 | 0.9979982 |
| QBD-post | 90 | 171 | 259.4 | 239.7525 | 3.0588406 | 0.08099945 | 0.9984914 |
| QBD-post | 141 | 230 | 400.2609 | 390.0505 | 2.8326251 | 0.13292025 | 0.9974179 |
| QBD-post | 84 | 166 | 273.6471 | 271.1439 | 2.987414 | 0.07784519 | 0.9982303 |
| QBD-post | 188 | 163 | 231.3333 | 205.9436 | 3.3781119 | 0.05268324 | 0.9988105 |
| QBD-post | 112 | 214 | 319.12 | 337.475 | 3.4597455 | 0.04673332 | 0.9978821 |
| QBD-post | 71 | 170 | 203.4444 | 223.6729 | 2.4452677 | 0.20882953 | 0.9987525 |
| QBD-post | 121 | 86 | 125 | 128.1347 | 1.9527422 | 0.21141933 | 0.9992167 |
| QBD-post | 88 | 120 | 195.25 | 186.1558 | 2.2419181 | 0.15102756 | 0.9987525 |
| QBD-post | 106 | 156 | 198 | 213.6035 | 1.8879089 | 0.26841665 | 0.9985784 |

Supplemtary table S3 The dominant and differential bacterial species at phylum, genus and species levels among preoperative, postoperative group of CC patients and healthy control group (Taxon:the name of the species; mean in group:the mean value of the relative abundance of a species in a group of samples(control: healthy control group, CC-Pre: the preoperative group of CC patients, CC-Post: the postoperative group of CC patients); Pvalue: statistical significance value of difference analysis; FDR: P value corrected by BH method; P.adj: Tukey HSD between CC-Pre group and CC-Post group, P value after multiple corrections.)

| Taxon | mean in control | mean in CC-Pre | mean in CC-Post | Pvalue | FDR | P.adj |
| --- | --- | --- | --- | --- | --- | --- |
| Phylum | | | | | | |
| Bacteroidetes | 0.224761615 | 0.038294664 | 0.222499337 | 4.85115E-12 | 1.35832E-10 | 2.57442E-11 |
| Proteobacteria | 0.071586931 | 0.258941351 | 0.100043795 | 1.94633E-11 | 2.72486E-10 | 4.79525E-10 |
| Verrucomicrobia | 0.042109106 | 0.04852465 | 0.016967774 | 0.063344187 | 0.197070804 | 0.056017486 |
| Fusobacteria | 0.004222516 | 0.01281589 | 0.007037584 | 0.282047084 | 0.607486028 | 0.391925196 |
| Actinobacteria | 0.042857385 | 0.067285667 | 0.059533135 | 0.437230623 | 0.771645026 | 0.81660285 |
| Firmicutes | 0.598222748 | 0.570959192 | 0.591246816 | 0.789627561 | 0.921232155 | 0.825337913 |
| Genus | | | | | | |
| Oscillibacter | 0.015837153 | 0.000633438 | 0.003807189 | 7.49945E-14 | 2.62481E-11 | 0.024143597 |
| Bacteroides | 0.125071322 | 0.024747247 | 0.14031303 | 6.71705E-09 | 3.91828E-07 | 8.18437E-09 |
| Escherichia/Shigella | 0.045093758 | 0.171477547 | 0.062126983 | 5.90637E-07 | 2.06723E-05 | 3.10458E-06 |
| Faecalibacterium | 0.048925815 | 0.012697423 | 0.055248277 | 1.60392E-06 | 5.10339E-05 | 1.57041E-06 |
| Streptococcus | 0.011189122 | 0.076311638 | 0.029897987 | 3.35813E-06 | 9.79455E-05 | 5.8973E-05 |
| Alistipes | 0.029736519 | 0.001921036 | 0.015305777 | 1.0805E-05 | 0.000273082 | 0.003403417 |
| Ruminococcus | 0.029163524 | 0.002943033 | 0.01619929 | 1.09233E-05 | 0.000273082 | 0.002303559 |
| Enterococcus | 0.020393312 | 0.154703535 | 0.040718237 | 1.74248E-05 | 0.000381168 | 6.68601E-05 |
| Clostridium IV | 0.023092675 | 0.004063465 | 0.007228927 | 8.51521E-05 | 0.001753131 | 0.513266816 |
| Erysipelotrichaceae incertae sedis | 0.001137529 | 0.021722357 | 0.006185862 | 0.000109349 | 0.00212623 | 0.000613108 |
| Klebsiella | 0.006821545 | 0.057675995 | 0.014907547 | 0.000239024 | 0.004331528 | 0.000675691 |
| Dorea | 0.015134811 | 0.007391949 | 0.020075557 | 0.002219934 | 0.028776925 | 0.00144463 |
| Parabacteroides | 0.025361688 | 0.00728488 | 0.018279895 | 0.009323435 | 0.083214653 | 0.044727127 |
| Prevotella | 0.009495522 | 0.001739709 | 0.032948781 | 0.01603307 | 0.121990754 | 0.01295171 |
| Blautia | 0.059978337 | 0.038467011 | 0.069277853 | 0.02030355 | 0.142124847 | 0.015794749 |
| Lachnospiracea incertae sedis | 0.046771643 | 0.038129224 | 0.059456805 | 0.049733109 | 0.255979236 | 0.038699574 |
| Clostridium XVIII | 0.008239527 | 0.018264698 | 0.026449999 | 0.05178468 | 0.262675913 | 0.267829085 |
| Akkermansia | 0.042072841 | 0.048521196 | 0.016868648 | 0.062020195 | 0.297357099 | 0.054788659 |
| Ruminococcus2 | 0.018318924 | 0.016793354 | 0.026310463 | 0.246342384 | 0.628740944 | 0.234038359 |
| Lactobacillus | 0.008229856 | 0.023657209 | 0.01026107 | 0.27353902 | 0.635318931 | 0.308939165 |
| Holdemanella | 0.017640759 | 0.007681728 | 0.010328421 | 0.299238049 | 0.638157924 | 0.809496994 |
| Peptostreptococcus | 0.008946704 | 0.012277088 | 0.006762311 | 0.325422344 | 0.650844689 | 0.29378621 |
| Clostridium XlVa | 0.006652306 | 0.012343747 | 0.00919141 | 0.343051936 | 0.67081141 | 0.528950688 |
| Bifidobacterium | 0.009806197 | 0.024805962 | 0.02846878 | 0.417783322 | 0.67081141 | 0.91980802 |
| Collinsella | 0.017164471 | 0.011293774 | 0.016175804 | 0.454859304 | 0.67081141 | 0.498968732 |
| Specics | | | | | | |
| Escherichia coli K-12 | 0.045084088 | 0.1714399 | 0.062114549 | 5.9126E-07 | 2.20586E-05 | 3.10765E-06 |
| Bacteroides uniformis | 0.021978115 | 0.001416773 | 0.01392527 | 7.5751E-06 | 0.000183696 | 0.000396422 |
| Eubacterium rectale ATCC33656 | 0.022289998 | 0.002122051 | 0.013989166 | 7.38042E-05 | 0.001556305 | 0.002264349 |
| Clostridium innocuum | 0.001021479 | 0.020469642 | 0.00543672 | 0.000152936 | 0.002747188 | 0.000705365 |
| uncultured organism | 0.261852781 | 0.206493463 | 0.295628243 | 0.000650669 | 0.008955375 | 0.000403186 |
| Streptococcus salivarius | 0.004064156 | 0.023443415 | 0.008963455 | 0.00369541 | 0.035845481 | 0.010837887 |
| Bacteroides fragilis 638R | 0.01627234 | 0.005231215 | 0.029437587 | 0.005167789 | 0.049144663 | 0.003419023 |
| Bacteroides stercoris | 0.009895652 | 0.000888678 | 0.014182582 | 0.006828767 | 0.061332448 | 0.004953152 |
| Bacteroides thetaiotaomicron | 0.009919829 | 0.004316979 | 0.015294725 | 0.032543438 | 0.159899412 | 0.024200326 |
| Clostridium ramosum | 0.002798489 | 0.0127216 | 0.008987978 | 0.128242816 | 0.438012435 | 0.5047605 |
| Collinsella aerofaciens | 0.01712337 | 0.010972565 | 0.016004147 | 0.428395357 | 0.715129157 | 0.477292836 |

Supplemtary table S4 The specific and differential bacterial species at phylum, class, order, family, genus and species levels among the preoperative group of CC patients with the five TCM sydrome types and healthy control group (Taxon:the name of the species; mean in group:the mean value of the relative abundance of a species in a group of samples(control: healthy control group, DHA-Pre: the preoperative group of CC patients with DHA sydrome type, SDD-Pre: the preoperative group of CC patients with SDD sydrome type, SKYD-Pre: the preoperative group of CC patients with SKYD sydrome type, LKYD-Pre: the preoperative group of CC patients with LKYD sydrome type, QBD-Pre: the preoperative group of CC patients with QBD sydrome type); LDA score: the effect value of each species with significant

difference by Linear discriminant analysis (LDA)(the greater the absolute value, the easier to distinguish among groups); with |LDA|>2 and P<0.05 is used as the difference screening threshold to obtain species with significant differences in relative abundance among groups; FDR: P value corrected by BH method through ANOVA analysis; ^*^ indicates that the taxons has the highest relative abundance in this group.

| Taxon | mean in Control | mean in DHA-pre | mean in SDD-pre | mean in SKYD-pre | mean in LKYD-pre | mean in QBD-pre | LDA score | P | FDR |
| --- | --- | --- | --- | --- | --- | --- | --- | --- | --- |
| Phylum | | | | | | | | | |
| Euryarchaeota | 0.010510957^*^ | 0.0021034 | 0.003850915 | 5.97314E-05 | 5.07717E-05 | 2.90124E-05 | 3.73136896 | 4.19E-05 | 8.60356E-05 |
| Verrucomicrobia | 0.041899975 | 0.018002205 | 0.139410468^*^ | 0.004160722 | 0.012231152 | 0.076263974 | 4.857147365 | 5.64E-06 | 0.01569658 |
| Fusobacteria | 0.004113719 | 0.003697764 | 0.036261653^*^ | 0.011092983 | 0.004598468 | 0.011957951 | 4.163137668 | 0.026286 | 0.12183916 |
| Proteobacteria | 0.071570007 | 0.22238941 | 0.178476655 | 0.5006195^*^ | 0.239180786 | 0.15512456 | 5.324772821 | 1.63E-08 | 2.79911E-12 |
| Class | | | | | | | | | |
| Gammaproteobacteria | 0.060044824 | 0.219198044 | 0.175476771 | 0.486207155^*^ | 0.233890855 | 0.153952781 | 3.553342769 | 7.63E-07 | 1.02478E-11 |
| Bacilli | 0.049900149 | 0.262367202 | 0.133165061 | 0.109235164 | 0.48642461^*^ | 0.414376297 | 5.3487244 | 2.35E-09 | 3.21468E-10 |
| Methanobacteria | 0.010510957^*^ | 0.0021034 | 0.003850915 | 5.97314E-05 | 5.07717E-05 | 2.90124E-05 | 3.731369122 | 4.19E-05 | 8.29629E-05 |
| Verrucomicrobiae | 0.041870962 | 0.018002205 | 0.139408534^*^ | 0.004160722 | 0.012231152 | 0.076263974 | 4.85714071 | 7.32E-06 | 0.018017481 |
| Fusobacteriia | 0.004113719 | 0.003697764 | 0.036261653^*^ | 0.011092983 | 0.004598468 | 0.011957951 | 4.162888857 | 0.02628646 | 0.150625335 |
| Negativicutes | 0.018693668 | 0.017626362 | 0.009581834 | 0.022726962 | 0.000824436 | 0.02632232^*^ | 4.102296329 | 0.00050484 | 0.692882571 |
| Order | | | | | | | | | |
| Enterobacteriales | 0.057549756 | 0.217230475 | 0.167933542 | 0.477781608^*^ | 0.232877838 | 0.152232989 | 5.314389632 | 1.07E-08 | 1.96549E-11 |
| Lactobacillales | 0.04901527 | 0.257677741 | 0.1311516 | 0.107909126 | 0.46476200^*^ | 0.401713989 | 5.324769217 | 5.15E-09 | 7.96863E-10 |
| Methanobacteriales | 0.010510957^*^ | 0.0021034 | 0.003850915 | 5.97314E-05 | 5.07717E-05 | 2.90124E-05 | 3.731328715 | 4.19E-05 | 0.000119835 |
| Verrucomicrobiales | 0.041870962 | 0.018002205 | 0.139408534^*^ | 0.004160722 | 0.012231152 | 0.076263974 | 4.85714071 | 7.32E-06 | 0.026025251 |
| Rhizobiales | 8.46196E-06 | 1.31875E-05 | 5.80248E-06 | 8.53306E-06 | 2.4177E-06 | 3.3848E-05^*^ | 3.946832835 | 0.01358384 | 0.044312616 |
| Fusobacteriales | 0.004113719 | 0.003697764 | 0.036261653^*^ | 0.011092983 | 0.004598468 | 0.011957951 | 4.162888583 | 0.02628646 | 0.188560604 |
| Selenomonadales | 0.018693668 | 0.017626362 | 0.009581834 | 0.022726962 | 0.000824436 | 0.02632232^*^ | 4.102296329 | 0.00050484 | 0.657688535 |
| Family | | | | | | | | | |
| Aerococcaceae | 3.62655E-06 | 0.000181987 | 9.09056E-05 | 8.3624E-05 | 0.000423098 | 0.00109119^*^ | 2.8098456 | 0.00165639 | 0.281698605 |
| Enterobacteriaceae | 0.057549756 | 0.217230475 | 0.167933542 | 0.477781608^*^ | 0.232877838 | 0.152232989 | 5.261441744 | 1.18E-06 | 1.96549E-11 |
| Enterococcaceae | 0.02036309 | 0.074924568 | 0.039590345 | 0.059146079 | 0.37612665^*^ | 0.291313682 | 5.261441744 | 1.18E-06 | 1.18679E-07 |
| Peptococcaceae_1 | 0.000650362 | 0.000654098^*^ | 9.67081E-06 | 0.000310604 | 0 | 3.86832E-05 | 2.595536643 | 8.93E-06 | 0.52182466 |
| Oxalobacteraceae | 0.000203087^*^ | 6.59373E-06 | 5.80248E-06 | 1.19463E-05 | 0 | 3.2236E-06 | 2.569809119 | 4.34E-11 | 1.35815E-06 |
| Methanobacteriaceae | 0.010510957^*^ | 0.0021034 | 0.003850915 | 5.97314E-05 | 5.07717E-05 | 2.90124E-05 | 3.731399179 | 4.19E-05 | 0.000171193 |
| Christensenellaceae | 0.003064437^*^ | 0.000242649 | 0.000317202 | 0.000228686 | 7.2531E-06 | 2.09534E-05 | 3.138809182 | 2.30E-10 | 0.002231462 |
| Burkholderiaceae | 5.92337E-05 | 0.0001055 | 4.44857E-05 | 4.09587E-05 | 4.8354E-05 | 0.00028207^*^ | 2.860983901 | 0.00733685 | 0.013435747 |
| Verrucomicrobiaceae | 0.041870962 | 0.018002205 | 0.139408534^*^ | 0.004160722 | 0.012231152 | 0.076263974 | 4.85714071 | 7.32E-06 | 0.026025251 |
| Streptococcaceae | 0.014890623 | 0.092504774 | 0.081903988 | 0.037335568 | 0.069496828 | 0.10004287^*^ | 4.59186125 | 0.00048075 | 0.029874115 |
| Actinomycetaceae | 0.000984004 | 0.00494398 | 0.00534989 | 0.001368703 | 0.006138544 | 0.01189187^*^ | 3.725564371 | 2.48E-05 | 0.116157452 |
| Clostridiaceae_1 | 0.019780424^*^ | 0.005693028 | 0.007904917 | 0.00169296 | 0.000614096 | 0.004350251 | 3.984457056 | 0.00280869 | 0.333803945 |
| Fusobacteriaceae | 0.00406053 | 0.003685896 | 0.036259719^*^ | 0.01108957 | 0.004598468 | 0.01192088 | 4.162988777 | 0.03115302 | 0.194776482 |
| Carnobacteriaceae | 0.000131765 | 0.001635245 | 0.0018684 | 0.001124658 | 0.00573721^*^ | 0.005011089 | 3.37210508 | 0.00012831 | 0.194776482 |
| Genus | | | | | | | | | |
| Escherichia/Shigella | 0.044980126 | 0.145062087 | 0.146344435 | 0.408609179^*^ | 0.012318189 | 0.10711868 | 5.296148909 | 1.28E-09 | 1.05304E-11 |
| Oscillibacter | 0.015931444^*^ | 0.001007522 | 0.000972883 | 0.00073043 | 9.18727E-05 | 0.000190193 | 3.864796538 | 1.69E-12 | 5.60333E-09 |
| Weissella | 0.00532257 | 0.017295357^*^ | 0.00055317 | 9.89835E-05 | 0.003568527 | 8.70373E-05 | 3.96210077 | 0.00035878 | 0.595705543 |
| Peptococcus | 0.000528268 | 0.000651461^*^ | 9.67081E-06 | 0.000310604 | 0 | 3.86832E-05 | 2.520150934 | 0.01607655 | 0.669561635 |
| Enterococcus | 0.02036309 | 0.074907424 | 0.039540056 | 0.059069282 | 0.37612423^*^ | 0.291304011 | 5.261439524 | 1.19E-06 | 3.01418E-07 |
| Oxalobacter | 0.000201878^*^ | 6.59373E-06 | 5.80248E-06 | 1.02397E-05 | 0 | 0 | 2.492334998 | 1.90E-12 | 3.64895E-06 |
| Sporobacter | 0.001670632^*^ | 0.000160887 | 3.86832E-05 | 4.26653E-05 | 2.4177E-06 | 9.67081E-06 | 2.937176027 | 7.10E-13 | 0.000181676 |
| Methanobrevibacter | 0.009814659^*^ | 0.001213247 | 0.003721326 | 5.97314E-05 | 5.07717E-05 | 2.90124E-05 | 3.704860782 | 4.02E-05 | 0.000181676 |
| Burkholderia | 7.2531E-06 | 3.29687E-05 | 2.51441E-05 | 1.87727E-05 | 2.65947E-05 | 0.00017408^*^ | 3.155338224 | 0.01719866 | 0.002144947 |
| Christensenella | 0.003064437^*^ | 0.000242649 | 0.000317202 | 0.000228686 | 7.2531E-06 | 2.09534E-05 | 3.139055535 | 2.30E-10 | 0.003551028 |
| Papillibacter | 0.000301004^*^ | 5.01124E-05 | 0 | 1.36529E-05 | 0 | 0 | 2.766662756 | 1.45E-10 | 0.012156881 |
| Butyricicoccus | 0.00220857 | 0.002711342^*^ | 0.000932266 | 0.001711733 | 3.86832E-05 | 0.001349077 | 3.064740484 | 4.68E-06 | 0.550736831 |
| Eggerthella | 0.002470891 | 0.003422146 | 0.015815636^*^ | 0.008181502 | 0.008461955 | 0.007618983 | 3.850071687 | 0.0016591 | 0.018007788 |
| Streptococcus | 0.011190331 | 0.089807938 | 0.081203822 | 0.034615227 | 0.069470233 | 0.09967055^*^ | 4.611557954 | 0.00013641 | 0.029904607 |
| Akkermansia | 0.041870962 | 0.017998249 | 0.139408534^*^ | 0.004160722 | 0.012231152 | 0.076260751 | 4.85714071 | 7.45E-06 | 0.034555556 |
| Barnesiella | 0.003978328^*^ | 0.000196493 | 0.000216626 | 4.77852E-05 | 2.4177E-06 | 6.4472E-06 | 3.286635341 | 1.30E-05 | 0.034555556 |
| Clostridium_IV | 0.02318213^*^ | 0.009208805 | 0.002373216 | 0.004860433 | 0.000154733 | 0.000930009 | 4.050486589 | 3.31E-10 | 0.038501317 |
| Hydrogenoanaerobacterium | 2.05505E-05^*^ | 1.31875E-06 | 0 | 0 | 0 | 0 | 3.482521461 | 0.00010316 | 0.065366101 |
| Anaerotruncus | 0.001236654 | 2.90124E-05 | 0.002319059^*^ | 0.000679232 | 0.000285289 | 1.6118E-05 | 3.040681277 | 9.79E-06 | 0.071957712 |
| Actinomyces | 0.000970707 | 0.00491233 | 0.005013346 | 0.00136017 | 0.006131291 | 0.01170168^*^ | 3.718046767 | 4.61E-05 | 0.138059474 |
| Fusobacterium | 0.00406053 | 0.003683258 | 0.036259719^*^ | 0.011086157 | 0.00459605 | 0.011916045 | 4.163016721 | 0.02787704 | 0.233163263 |
| Granulicatella | 0.000131765 | 0.001635245 | 0.0018684 | 0.001124658 | 0.00573479^*^ | 0.005007866 | 3.371892653 | 0.00014296 | 0.235485024 |
| Odoribacter | 0.013886068^*^ | 0.001701183 | 0.002431241 | 0.003442238 | 0.000282871 | 1.45062E-05 | 3.875550687 | 1.48E-07 | 0.235485024 |
| Anaerobacterium | 0.007276072^*^ | 0.001300284 | 3.86832E-06 | 5.11984E-06 | 7.2531E-06 | 1.28944E-05 | 3.526211136 | 1.28E-07 | 0.410747813 |
| Lactococcus | 0.003700292^*^ | 0.002696836 | 0.000700166 | 0.002720341 | 2.65947E-05 | 0.000372326 | 3.329128729 | 0.00351878 | 0.815233758 |
| Bilophila | 0.002308905 | 0.001320065 | 0.001241731 | 0.000679232 | 0.00284564^*^ | 0.00038522 | 3.170630634 | 7.47E-05 | 0.743788363 |
| Abiotrophia | 3.62655E-06 | 0.000170118 | 9.09056E-05 | 8.3624E-05 | 0.000423098 | 0.00109119^*^ | 2.81533593 | 0.00231282 | 0.332983415 |
| Finegoldia | 4.23098E-05 | 0.000338918 | 0.000305597 | 0.000225273 | 4.35186E-05 | 0.00394085^*^ | 3.363502678 | 0.00653186 | 0.662456672 |
| Ralstonia | 5.19806E-05 | 3.56061E-05 | 1.93416E-05 | 1.70661E-05 | 2.17593E-05 | 0.00010316^*^ | 3.127542631 | 0.01030385 | 0.410747813 |
| Eisenbergiella | 0.001453039 | 0.00013715 | 0.001584078^*^ | 0.001413075 | 0.00014748 | 0 | 2.947629469 | 8.44E-08 | 0.207515913 |
| Campylobacter | 0.000182536^*^ | 5.80248E-05 | 1.1605E-05 | 1.19463E-05 | 2.4177E-06 | 9.67081E-05 | 3.472017364 | 0.00053767 | 0.768130023 |
| Pyramidobacter | 0.001431279^*^ | 0.000110775 | 0.000177943 | 0.000923278 | 4.8354E-06 | 8.059E-06 | 2.924830647 | 0.01935909 | 0.669561635 |
| Species | | | | | | | | | |
| Escherichia coli K-12 | 0.044968038 | 0.145013293 | 0.146319291 | 0.408515315^*^ | 0.012318189 | 0.107115457 | 5.296054618 | 1.28E-09 | 1.45965E-11 |
| Methanobrevibacter smithii | 0.009814659^*^ | 0.000662011 | 0.003719392 | 5.97314E-05 | 5.07717E-05 | 2.90124E-05 | 3.70493414 | 2.69E-05 | 0.000175289 |
| Clostridium sp._YIT_12070 | 3.2639E-05 | 6.59373E-06 | 5.02882E-05^*^ | 0 | 0 | 1.6118E-06 | 3.537305907 | 2.91E-05 | 0.506011972 |
| Clostridium symbiosum | 0.000485958 | 0.000164843 | 0.004409887^*^ | 0.000849893 | 9.91258E-05 | 0.00032236 | 3.325379525 | 0.00245818 | 0.006059094 |
| Eggerthella lenta | 0.002468473 | 0.003420828 | 0.015815636^*^ | 0.008181502 | 0.008461955 | 0.007617371 | 3.850148686 | 0.00165254 | 0.018581359 |
| Lactobacillus fermentum | 3.2639E-05 | 0.000174075^*^ | 2.90124E-05 | 8.53306E-06 | 4.8354E-06 | 1.12826E-05 | 2.938423126 | 0.03124168 | 0.032578035 |
| Eubacteriumeligens ATCC_27750 | 0.00048354 | 0.000498486^*^ | 3.86832E-06 | 8.87439E-05 | 4.8354E-06 | 5.80248E-05 | 2.786285399 | 3.11E-08 | 0.148536766 |
| Alistipes indistinctus | 0.001460292^*^ | 0.000193856 | 3.67491E-05 | 6.31447E-05 | 1.45062E-05 | 0 | 2.828483788 | 7.81E-08 | 0.002222206 |
| Clostridium scindens | 0.002360885 | 0.00180932 | 0.007448455* | 0.002251022 | 0.002064717 | 1.77298E-05 | 3.641579357 | 4.26E-06 | 0.211883978 |
| bacterium P1C10 | 3.62655E-05^*^ | 1.31875E-06 | 3.86832E-06 | 0 | 0 | 0 | 3.405796774 | 7.27E-05 | 0.001384417 |
| Catabacter hongkongensis | 7.01133E-05 | 0.000983785^*^ | 0.000179877 | 6.99711E-05 | 3.14301E-05 | 6.76956E-05 | 2.986919968 | 0.00138748 | 0.722496225 |
| Streptococcus mutans | 3.14301E-05 | 0.000274299^*^ | 5.80248E-05 | 3.0719E-05 | 5.80248E-05 | 0.000230488 | 2.874495534 | 0.01883910 | 0.570786246 |
| Clostridium leptum | 0.000415845 | 5.14311E-05 | 0.000127655 | 0.001402836^*^ | 0 | 0 | 3.033997915 | 5.86E-07 | 0.722047396 |
| Lachnospira pectinoschiza | 0.000246606 | 0.000151656 | 3.86832E-06 | 0.000646806^*^ | 0 | 1.6118E-06 | 2.878936835 | 1.98E-06 | 0.722047396 |
| Blautia producta | 0.000991258 | 0.000847954 | 0.002794863 | 0.003339841^*^ | 0.001133902 | 0.001358748 | 3.22120323 | 0.00191524 | 0.722047396 |
| Parabacteroides goldsteinii | 0.000968289 | 1.84624E-05 | 8.51031E-05 | 0.000426653 | 0.000427933 | 0.00137809* | 2.927117183 | 1.18E-05 | 0.722047396 |
| Corynebacterium tuberculostearicum | 2.4177E-06 | 1.31875E-05 | 6.38273E-05 | 1.36529E-05 | 9.67081E-06 | 0.00643753^*^ | 3.699527335 | 0.00089646 | 0.701167227 |
| Lactobacillus iners | 1.57151E-05 | 2.76937E-05 | 1.1605E-05 | 2.90124E-05 | 0 | 3.5459E-05^*^ | 3.57770294 | 0.00251635 | 0.834083819 |
| Alistipes sp__NML05A004 | 0.000604425^*^ | 9.23122E-06 | 0 | 0 | 0 | 1.6118E-06 | 2.609142644 | 4.50E-14 | 1.02535E-06 |
| Parabacteroides distasonis ATCC_8503 | 0.014873699^*^ | 0.008831644 | 0.003847047 | 0.001938712 | 0.003464566 | 0.000372326 | 3.825655457 | 2.79E-05 | 0.722047396 |
| Lactococcus garvieae | 0.003347308^*^ | 0.001015435 | 0.000522224 | 0.002592345 | 1.45062E-05 | 2.09534E-05 | 3.227829936 | 0.004217555 | 0.78757595 |

Supplemtary table S5 The specific and differential bacterial species at phylum, class, order, family, genus and species levels among the postoperative group of CC patients with the five TCM sydrome types and healthy control group (Taxon:the name of the species; mean in group:the mean value of the relative abundance of a species in a group of samples(control: healthy control group, DHA-Post: the postoperative group of CC patients with DHA sydrome type, SDD-Post: the postoperative group of CC patients with SDD sydrome type, SKYD-Post: the postoperative group of CC patients with SKYD sydrome type, LKYD-Post: the postoperative group of CC patients with LKYD sydrome type, QBD-Post: the postoperative group of CC patients with QBD sydrome type); LDA score: the effect value of each species with significant

difference by Linear discriminant analysis (LDA) (the greater the absolute value, the easier to distinguish among groups); with |LDA|>2 and P<0.05 is used as the difference screening threshold to obtain species with significant differences in relative abundance among groups; FDR: P value corrected by BH method through ANOVA analysis; ^*^ indicates that the taxons has the highest relative abundance in this group.

| Taxon | Control | DHA-post | SDD-post | SKYD-post | LKYD-post | QBD-post | LDA score | P | FDR |
| --- | --- | --- | --- | --- | --- | --- | --- | --- | --- |
| Phylum | | | | | | | | | |
| Bacteroidetes | 0.225293357 | 0.488867433 | 0.10056553 | 0.03070804 | 0.50325884^*^ | 0.26761999 | 5.372138527 | 5.12E-12 | 2.72013E-16 |
| Firmicutes | 0.597336913 | 0.400466479 | 0.7538124^*^ | 0.75299693 | 0.272438707 | 0.50673561 | 5.391623718 | 2.55E-10 | 1.14848E-13 |
| Euryarchaeota | 0.010623508^*^ | 0.001839878 | 0.00104078 | 0.00059325 | 0.002343241 | 0.00154999 | 3.720978278 | 3.16E-05 | 9.29299E-06 |
| Actinobacteria | 0.043005171 | 0.024463007 | 0.07499844 | 0.102831^*^ | 0.012900846 | 0.0399642 | 4.627304133 | 3.06E-05 | 0.000390095 |
| Fusobacteria | 0.004254177 | 0.007105663 | 0.00058732 | 0.00214569 | 0.01964634^*^ | 0.01324980 | 3.986893123 | 0.011110142 | 0.16902213 |
| Class | | | | | | | | | |
| Clostridia | 0.485072593 | 0.29464309 | 0.58874027 | 0.6361950^*^ | 0.175552181 | 0.20622514 | 5.364722435 | 2.30E-13 | 9.60356E-21 |
| Bacteroidia | 0.225190931 | 0.488789325 | 0.10053855 | 0.03068417 | 0.50323931^*^ | 0.26759613 | 5.37214045 | 5.12E-12 | 2.1599E-16 |
| Methanobacteria | 0.010623508^*^ | 0.001839878 | 0.00103974 | 0.00059325 | 0.002341072 | 0.00154999 | 3.720318694 | 3.00E-05 | 1.4923E-05 |
| Bacilli | 0.049605211 | 0.053356476 | 0.06753450 | 0.03546426 | 0.050529399 | 0.2486834^*^ | 5.023646799 | 0.000206921 | 0.000211624 |
| Actinobacteria | 0.043003182 | 0.024463007 | 0.07499844 | 0.1028265^*^ | 0.012900846 | 0.03996288 | 4.62728069 | 3.06E-05 | 0.000501463 |
| Fusobacteriia | 0.004254177 | 0.007105663 | 0.00058732 | 0.00214569 | 0.01964634^*^ | 0.01324980 | 3.986708671 | 0.011110142 | 0.165715048 |
| Order | | | | | | | | | |
| Clostridiales | 0.485072593 | 0.29464309 | 0.58874027 | 0.6361950^*^ | 0.175552181 | 0.206225139 | 5.364722435 | 2.30E-13 | 1.36584E-20 |
| Bacteroidales | 0.225190931 | 0.488789325 | 0.10053855 | 0.03068417 | 0.50323931^*^ | 0.26759613 | 5.37214045 | 5.12E-12 | 3.07186E-16 |
| Methanobacteriales | 0.010623508^*^ | 0.001839878 | 0.00103974 | 0.00059325 | 0.002341072 | 0.00154999 | 3.722542808 | 3.00E-05 | 2.12239E-05 |
| Lactobacillales | 0.048730111 | 0.050423085 | 0.04846737 | 0.03133538 | 0.046836624 | 0.2415420^*^ | 5.017311353 | 0.00051716 | 0.000179191 |
| Burkholderiales | 0.003434765 | 0.002115426 | 0.00420255 | 0.00131379 | 0.01896724^*^ | 0.00125033 | 3.893059658 | 0.032490769 | 0.01556958 |
| Fusobacteriales | 0.004254177 | 0.007105663 | 0.00058732 | 0.00214569 | 0.01964634^*^ | 0.01324980 | 3.98639804 | 0.011110142 | 0.186053803 |
| Family | | | | | | | | | |
| Lachnospiraceae | 0.198892204 | 0.116908223 | 0.28261181 | 0.4568030^*^ | 0.105246257 | 0.09982365 | 5.259173319 | 1.66E-12 | 7.75222E-18 |
| Clostridiaceae_1 | 0.019643994^*^ | 0.005760469 | 0.01668465 | 0.01124219 | 0.000798438 | 0.00166534 | 3.984457056 | 0.00280869 | 0.446162409 |
| Carnobacteriaceae | 0.000138226 | 0.0006509 | 0.00045969 | 0.00087851 | 0.00134086^*^ | 0.00129541 | 2.78952352 | 0.000268665 | 0.249508576 |
| Aerococcaceae | 6.96102E-06 | 1.08483E-05 | 2.6979E-05 | 6.0234E-05 | 0.00014537^*^ | 0.00014452 | 3.014629855 | 0.000517667 | 0.302252103 |
| Leuconostocaceae | 0.005408711 | 0.00189412 | 0.0068216^*^ | 0.00204682 | 4.77327E-05 | 0.00063776 | 3.492267173 | 0.00345793 | 0.709220536 |
| Ruminococcaceae | 0.186450875 | 0.138186158 | 0.2352091^*^ | 0.14014547 | 0.045452376 | 0.05975073 | 4.964673015 | 1.53E-09 | 1.33998E-07 |
| Methanobacteriaceae | 0.010623508^*^ | 0.001839878 | 0.00103974 | 0.00059325 | 0.002341072 | 0.00154999 | 3.720376779 | 3.00E-05 | 3.2582E-05 |
| Rikenellaceae | 0.029637033 | 0.06290085^*^ | 0.00891875 | 0.00057620 | 0.011275765 | 0.01378945 | 4.401115819 | 3.81E-09 | 0.000149416 |
| Enterococcaceae | 0.020294352 | 0.003460621 | 0.00963993 | 0.00762703 | 0.034031243 | 0.1466415^*^ | 4.820068726 | 9.08E-05 | 0.000808183 |
| Christensenellaceae | 0.003087709^*^ | 0.000232154 | 0.00016188 | 0.00011024 | 0.000171404 | 2.917E-05 | 3.21075421 | 1.13E-08 | 0.001476016 |
| Oxalobacteraceae | 0.000212808^*^ | 0.00013018 | 5.49964E-0 | 3.40948E-0 | 4.12237E-05 | 2.917E-05 | 2.569809118 | 4.34E-11 | 0.01835308 |
| Acidaminococcaceae | 0.007593477 | 0.011740074 | 0.00304244 | 0.00079781 | 0.008134085 | 0.0147998^*^ | 3.772143931 | 0.000362817 | 0.311979479 |
| Fusobacteriaceae | 0.004215394 | 0.007101323 | 0.00058524 | 0.00213319 | 0.01923411^*^ | 0.01324582 | 3.977641392 | 0.017250873 | 0.266882391 |
| Genus | | | | | | | | | |
| Odoribacter | 0.013764916^*^ | 0.007676286 | 0.00230051 | 0.00010228 | 0.004593187 | 0.01226996 | 3.875550687 | 1.48E-07 | 0.757212126 |
| Lactococcus | 0.003673429^*^ | 0.000774571 | 0.00139566 | 0.00029889 | 1.08483E-05 | 0.00028507 | 3.329128729 | 0.003518781 | 0.760844802 |
| Lachnospiracea_incertae_sedis | 0.046952068 | 0.021026253 | 0.06724292 | 0.1238436^*^ | 0.019134302 | 0.02287589 | 4.738135014 | 2.08E-08 | 1.38614E-07 |
| Abiotrophia | 3.97772E-06 | 1.08483E-05 | 2.6979E-05 | 6.0234E-05 | 0.00014318^*^ | 0.00013524 | 2.968787827 | 0.001532046 | 0.45077745 |
| Blautia | 0.059796142 | 0.030742026 | 0.08603299 | 0.1418263^*^ | 0.005406813 | 0.02527181 | 4.845637529 | 9.52E-09 | 3.75784E-07 |
| Faecalibacterium | 0.048749006 | 0.056409199 | 0.1124748^*^ | 0.05138084 | 0.006491647 | 0.01533811 | 4.724456594 | 1.98E-07 | 0.00020028 |
| Anaerostipes | 0.003005171 | 0.000926448 | 0.00656325 | 0.0184282^*^ | 0.000216967 | 0.00074649 | 3.964885147 | 8.92E-10 | 0.00020028 |
| Oscillibacter | 0.015910899^*^ | 0.00934693 | 0.00306735 | 0.00125355 | 0.008244739 | 0.00133254 | 3.873330762 | 5.66E-10 | 0.000380667 |
| Campylobacter | 0.000186953^*^ | 4.12237E-05 | 2.0753E-06 | 1.5911E-05 | 7.59384E-05 | 1.4585E-05 | 3.472017364 | 0.00053767 | 0.757212126 |
| Parabacteroides | 0.02548031 | 0.034742894 | 0.00578915 | 0.00127287 | 0.05510957^*^ | 0.02180589 | 4.423114041 | 6.43E-09 | 0.000915141 |
| Enterococcus | 0.020294352 | 0.003460621 | 0.00962540 | 0.00762473 | 0.034029074 | 0.1460236^*^ | 4.818458442 | 0.000180555 | 0.001242522 |
| Romboutsia | 0.00844471 | 0.003360816 | 0.0227716^*^ | 0.00912376 | 0.000644391 | 0.00279767 | 4.030020584 | 3.12E-07 | 0.001912168 |
| Christensenella | 0.003087709^*^ | 0.000232154 | 0.00016188 | 0.00011024 | 0.000171404 | 2.917E-05 | 3.139055535 | 2.30E-10 | 0.001930856 |
| Sporobacter | 0.001698488^*^ | 0.001282274 | 0.00067033 | 2.1593E-05 | 0.000273378 | 1.1933E-05 | 2.937176027 | 7.10E-13 | 0.005019817 |
| Clostridium_XVIII | 0.008209029 | 0.011236711 | 0.02140500 | 0.0551995^*^ | 0.009381645 | 0.01888491 | 4.382227723 | 9.38E-05 | 0.006463525 |
| Adlercreutzia | 0.002884845 | 0.000318941 | 0.0037792^*^ | 0.00012047 | 1.3018E-05 | 0.00010872 | 3.309923234 | 2.66E-08 | 0.014683497 |
| Oxalobacter | 0.000209825 | 0.00013018 | 5.1883E-05 | 3.0685E-05 | 4.12237E-05 | 2.6518E-05 | 2.492334998 | 1.90E-12 | 0.021190053 |
| Collinsella | 0.017209626 | 0.004701671 | 0.0254021 | 0.0319161^*^ | 0.003347798 | 0.00069610 | 4.115176784 | 0.000513062 | 0.024672397 |
| Holdemanella | 0.017652148 | 0.014102842 | 0.0286064^*^ | 0.00046483 | 0.003169885 | 0.00080748 | 4.13814187 | 0.000222979 | 0.081981467 |
| Gemmiger | 0.022023667 | 0.016914732 | 0.04632666 | 0.0494397^*^ | 0.002245606 | 0.01052108 | 4.400769811 | 5.79E-06 | 0.243680659 |
| Coprococcus | 0.008502387 | 0.008194836 | 0.01228391 | 0.0241948^*^ | 0.006200911 | 0.00518032 | 3.904391223 | 0.003024474 | 0.269422695 |
| Fusobacterium | 0.004214399 | 0.007101323 | 0.00058421 | 0.00206728 | 0.01923411^*^ | 0.01089764 | 3.977852854 | 0.016198926 | 0.269422695 |
| Weissella | 0.005302307 | 0.001753092 | 0.0065985^*^ | 0.00178998 | 4.77327E-05 | 0.00047998 | 3.476286723 | 0.00519831 | 0.757212126 |
| Papillibacter | 0.000307279^*^ | 3.2545E-05 | 0.00010377 | 6.8189E-05 | 0 | 1.3259E-06 | 2.81252789 | 4.83E-08 | 0.307487961 |
| Clostridium_IV | 0.023285601^*^ | 0.011293122 | 0.00974473 | 0.00719173 | 0.005736602 | 0.00223283 | 4.050486589 | 3.31E-10 | 0.347610467 |
| Granulicatella | 0.000138226 | 0.0006509 | 0.00045969 | 0.00087396 | 0.00134086^*^ | 0.00129409 | 2.791608535 | 0.000281613 | 0.304633012 |
| Phascolarctobacterium | 0.007436356 | 0.011620742 | 0.00282661 | 0.000769406 | 0.008090692 | 0.01479846^*^ | 3.771163011 | 0.000194244 | 0.398151608 |
| Anaerobacterium | 0.007306086^*^ | 0.002356259 | 0.00240635 | 2.2729E-06 | 1.3018E-05 | 1.0607E-05 | 3.526211136 | 1.28E-07 | 0.45077745 |
| Barnesiella | 0.003915076^*^ | 0.002475591 | 0.00060289 | 8.2964E-05 | 0.000101974 | 4.7733E-05 | 3.286635341 | 1.30E-05 | 0.057363654 |
| Hydrogenoanaerobacterium | 2.78441E-05^*^ | 0 | 2.0753E-06 | 0 | 0 | 0 | 3.482521461 | 0.000103163 | 0.001930856 |
| Pyramidobacter | 0.001454853^*^ | 6.29204E-05 | 2.3866E-05 | 3.8641E-05 | 0 | 1.5911E-05 | 2.924830647 | 0.019359093 | 0.45077745 |
| Methanobrevibacter | 0.009953262^*^ | 0.001798655 | 0.00079797 | 0.00059325 | 0.002341072 | 0.00154999 | 3.704860782 | 4.02E-05 | 0.000163732 |
| Oxalobacter | 0.000209825^*^ | 0.00013018 | 5.1883E-05 | 3.0685E-05 | 4.12237E-05 | 2.6518E-05 | 2.492334998 | 1.90E-12 | 0.021190053 |
| Species | | | | | | | | | |
| Methanobrevibacter smithii | 0.009953262^*^ | 0.001796485 | 0.000797966 | 0.000593249 | 0.002341072 | 0.001549987 | 3.70493414 | 2.69E-05 | 0.000225992 |
| Parabacteroides distasonis_ATCC_8503 | 0.014799125^*^ | 0.006871339 | 0.00155339 | 0.00062279 | 0.011596876 | 0.00876823 | 3.825655457 | 2.79E-05 | 0.535080838 |
| Parabacteroides merdae | 0.0066428 | 0.01588197 | 0.00249767 | 0.00040573 | 0.026680408^*^ | 0.005517104 | 4.108412593 | 2.15E-08 | 0.001422259 |
| Adlercreutzia equolifaciens | 0.002884845 | 0.000318941 | 0.003779184^*^ | 0.000120468 | 1.3018E-05 | 0.000108724 | 3.309746869 | 2.66E-08 | 0.024634517 |
| bacterium P1C10 | 2.8839E-05^*^ | 2.16967E-06 | 1.0377E-06 | 2.2729E-06 | 0 | 0 | 3.405796774 | 7.27E-05 | 0.024634517 |
| Ruminococcus callidus | 0.002645187 | 0.002946409 | 0.0046332^*^ | 0.00042732 | 1.9527E-05 | 1.3259E-06 | 3.386130185 | 1.48E-06 | 0.044757153 |
| Collinsella aerofaciens | 0.017169849 | 0.004653938 | 0.0253938 | 0.0312831^*^ | 0.003345628 | 0.00068682 | 4.101725603 | 0.000314605 | 0.045259608 |
| Coprococcus catus | 0.002215593 | 0.001989586 | 0.0026689^*^ | 0.00150472 | 0.001056628 | 0.00015911 | 3.105586959 | 3.14E-06 | 0.101793779 |
| Blautia producta | 0.000968576 | 0.000761553 | 0.00245719 | 0.0053211^*^ | 0.000340638 | 0.00090162 | 3.410066638 | 4.44E-05 | 0.187157388 |
| Alistipes indistinctus | 0.001406126^*^ | 0.001095682 | 0.00032375 | 3.5231E-05 | 0.001034932 | 0.00032485 | 2.828483788 | 7.81E-08 | 0.187488168 |
| Bacteroides thetaiotaomicron | 0.00981603 | 0.021223693 | 0.00597385 | 0.00205932 | 0.03740508^*^ | 0.02544948 | 4.236793681 | 2.33E-05 | 0.267512602 |
| Lactococcus garvieae | 0.003327367^*^ | 0.000282057 | 0.00047733 | 0.00012615 | 6.509E-06 | 0.00023601 | 3.227829936 | 0.004217555 | 0.757292563 |
| Alistipes sp__NML05A004 | 0.000627486^*^ | 0.000136689 | 0.00053544 | 3.4095E-06 | 3.68844E-05 | 4.7733E-05 | 2.819939156 | 1.45E-07 | 0.505618173 |
